# Development of a quantitative prediction algorithm for target organ-specific similarity of human pluripotent stem cell-derived organoids and cells

**DOI:** 10.1038/s41467-021-24746-w

**Published:** 2021-07-23

**Authors:** Mi-Ok Lee, Su-gi Lee, Cho-Rok Jung, Ye Seul Son, Jae-Woon Ryu, Kwang Bo Jung, Jun-Ho Ahn, Jung-Hwa Oh, Hyang-Ae Lee, Jung Hwa Lim, Janghwan Kim, Insu Jang, Jinhyuk Choi, Jaeeun Jung, Kunhyang Park, Byungwook Lee, Dae-Soo Kim, Mi-Young Son, Hyun-Soo Cho

**Affiliations:** 1 Korea Research Institute of Bioscience and Biotechnology, Daejeon, Republic of Korea; 2 Korea University of Science and Technology, Daejeon, Republic of Korea; 3 Korea Institute of Toxicology (KIT), Daejeon, Republic of Korea

**Keywords:** Bioinformatics, Biological models, Computational models, Pluripotent stem cells, Computational science

## Abstract

Human pluripotent stem cell (hPSC)-derived organoids and cells have similar characteristics to human organs and tissues. Thus, in vitro human organoids and cells serve as a superior alternative to conventional cell lines and animal models in drug development and regenerative medicine. For a simple and reproducible analysis of the quality of organoids and cells to compensate for the shortcomings of existing experimental validation studies, a quantitative evaluation method should be developed. Here, using the GTEx database, we construct a quantitative calculation system to assess similarity to the human organs. To evaluate our system, we generate hPSC-derived organoids and cells, and detected organ similarity. To facilitate the access of our system by researchers, we develop a web-based user interface presenting similarity to the appropriate organs as percentages. Thus, this program could provide valuable information for the generation of high-quality organoids and cells and a strategy to guide proper lineage-oriented differentiation.

## Abstract (web summary)

Quantitative methods to assess the quality of hPSC-derived organoids have not been developed. Here they present a prediction algorithm to assess the transcriptomic similarity between hPSC-derived organoids and the corresponding human target organs and perform validation on lung bud organoids, antral gastric organoids, and cardiomyocytes.

## Introduction

Animal models have served as essential tools for elucidating the pathogenesis of human disease and investigating potential therapeutic targets before entering clinical trials. However, animal models cannot completely reproduce human pathophysiology and have frequently failed to predict human responses because of various human-specific characteristics not present in animal species, such as immune-related responses and pharmacokinetics^1,2^. Human primary cells are considered the gold standard in vitro models for studying human physiology, disease, and drug response^3,4^. However, the isolation and in vitro expansion of primary cells are difficult, limiting their use in research for human disease and drug development. Therefore, robust and representative in vitro human organ/tissue models are urgently needed to overcome these limitations.

Recent stem cell biology studies have focused on the in vitro generation of tissue-specific functional cells or tissue analogs by inducing transitions between cellular states^5,6^. The most common method is to differentiate human pluripotent stem cells (hPSCs) into tissue-specific cells by regulating developmental signaling^5^. The development of stem cell differentiation technology has led to the efficient differentiation of hPSCs into various cell types, such as neurons^7^, cardiomyocytes (CMs)^8^, hepatocytes^9^, and lung airway epithelial cells^10^. Furthermore, advances in three-dimensional (3D) culture systems have enabled the development of complex organotypic models using 3D organoids that differentiate from stem cells into tissue-like miniature analogs that recapitulate complex tissue-specific cell compositions, architectures, and functions^11–13^. These 3D organoid technologies provide an opportunity to study human development and disease in depth by assessing cellular interactions, location, and structural changes^14,15^. Overall, technologies to manipulate cell fate provide various options that can produce in vitro models with similar properties to the corresponding tissues, and these strategies can be broadly applicable for in vitro disease modeling, especially human infectious disease modeling, an important topic recently, drug development, and cell-based therapies. However, multiple studies on the in vitro generation of tissue-specific cells or organoids from hPSCs have demonstrated critical limitations of current technology, including immature characteristics^16,17^, variation in quality^18,19^, and regional specificity^20,21^. Transcriptome analysis of diverse tissue organoids demonstrated that organoids from hPSCs have fetus-like characteristics even in long-term culture^19,22,23^. Thus, additional maturation methods must be developed to obtain advanced in vitro human models that more closely mimic human tissue. Moreover, heterogeneous production critically limits their utility in various biomedical applications^18,19^.

Currently, the development of tissue-specific differentiation methods and the quality control of differentiated cells/organoids rely on the analysis of tissue-specific markers by histology and gene expression analysis. Evaluating differentiation status using key tissue-specific markers can be an efficient strategy for the design and optimization of differentiation methods, but evaluating the similarity between human tissue and differentiated cells/organoids is difficult because experimental validation is laborious and time consuming. Although clustering analysis of the global transcriptome provides insight into lineage markers and the molecular similarity of differentiated cells or organoids to their counterpart human tissue, this method does not provide a quantitative and standardized assessment of the similarity between these structures.

Previously, we developed a quantitative prediction system to assess the similarity to the liver (LiGEP; Liver-specific Gene Expression Panel) of hPSC-derived hepatocytes and liver organoids to improve qualitative quality assessment. Using the LiGEP algorithm, we can calculate the similarity between liver organoids or differentiated hepatocytes and human liver tissue, providing researchers with valuable information for generating high-quality liver organoids and hepatocytes^24,25^. However, because the LiGEP algorithm can calculate similarity only to the liver, it is necessary to expand our study to other human organs. Additionally, we need to build a web-based analytics platform that is readily available to researchers.

Here, we developed quantitative calculation systems to assess organ-specific similarity based on organ-specific gene expression panels (Organ-GEP) using the GTEx public database (8,555 samples, 53 tissues), including a lung-specific gene expression panel (LuGEP), a stomach-specific gene expression panel (StGEP), and a heart-specific gene expression panel (HtGEP) and an analytical algorithm for direct comparison to human organs. We validated this analytical system with in-house RNA-seq data on 20 total RNA samples from different tissues. Moreover, we obtained the similarity (%) of hPSC-derived lung bud organoids (LBOs), gastric organoids (GOs) and CMs to the corresponding human organs. Finally, we developed a web-based user interface named the Web-based Similarity Analytics System (W-SAS; https://www.kobic.re.kr/wsas/) to provide an analytical algorithm to calculate similarity (percentage) and gene expression patterns for direct comparison to human target organs (liver, lung, stomach, and heart). Thus, our quantitative calculation system and W-SAS can provide a useful platform for evaluating and improving the quality of differentiated organoids/cells.

## Results

### Development of a quantitative calculation system to assess the similarity of hPSC-derived organoids and cells to organs

**Fig. 1:**
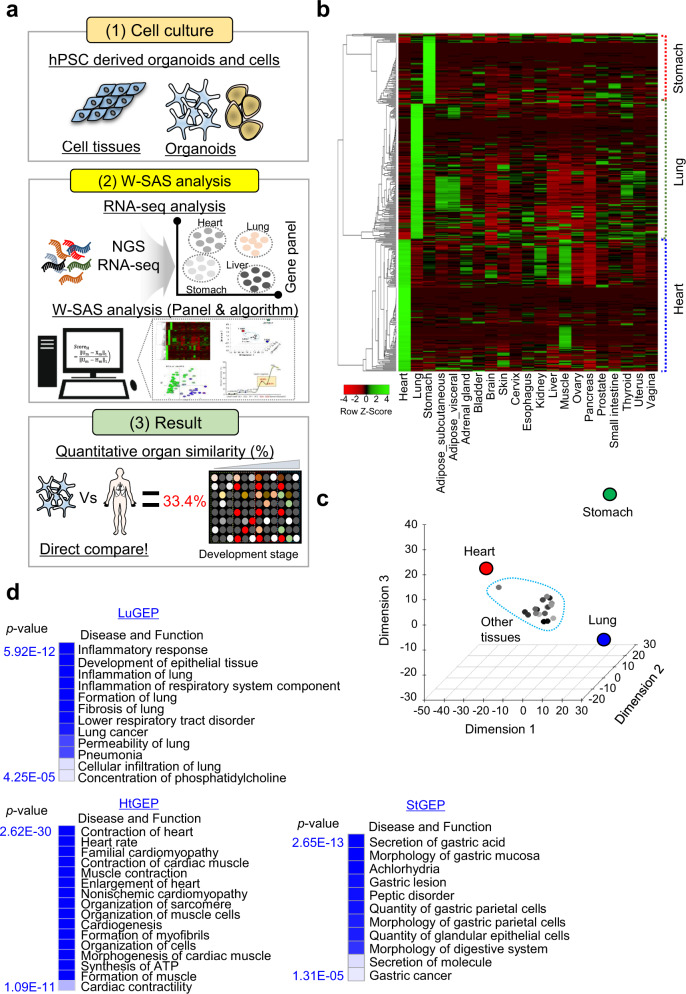
Construction of Organ-GEPs. **a** Schematic summary of the W-SAS system. **b** A heatmap representing the gene expression in Organ-GEPs (149 LuGEP, 73 StGEP, 144 HtGEP) as a percentage of samples from 21 tissues derived from the GTEx portal. **c** MDA plot analysis with RNA-seq results of Organ-GEP derived from the GTEx portal. **d** Disease and biofunctional analysis using Ingenuity Pathway Knowledge Base (IPA). The top regulatory diseases and biofunctions are selected, and the significance is displayed with a heatmap.

To assess the similarity of hPSC-derived organoids and cells to human organs at a quantitative level, in this study, we developed the W-SAS program for the quantitative assessment of the organ-specific similarity and quality of hPSC-derived organoids and cells. First, the researcher performs RNA-seq analysis. Using raw RNA-seq data (TPM, FPKM/RPKM values), the W-SAS program (organ-specific panels and algorithms) can calculate the similarity to the appropriate organ and provide a quantitative organ similarity score (%) and information on the gene expression patterns in organ-specific panels, directly comparing the target organs to the hPSC-derived organoids and cells. Using our system, researchers can obtain important information for the quality control of hPSC-derived organoids and cells (Fig. 1a).

### Construction of Organ-GEP to calculate the similarity of hPSC-derived organoids and cells to organs

To select organ-specific genes for each tissue (heart, lung, stomach), a three-step analysis was performed (t-test, confidence interval, quantile comparison). Step 1: Gene selection was performed by comparing the mean and variance between heart, lung or stomach tissues and the remaining tissues. We performed paired t-tests to identify differentially expressed genes between two tissues (the heart, lung, or stomach vs. one of 42 tissues) for all possible cases. We defined a set of tissue-specific genes through the intersection of genes that showed a significant difference (*p*-value < 0.05) in the *t*-test results. We defined 2843 heart-specific genes, 1049 lung-specific genes and 466 stomach-specific genes. Step 2: Since the genes acquired in the first step were chosen based on the difference in the mean and the variance between tissues, these genes were not only expressed uniquely in the particular tissue but also showed large variances in other tissues. Therefore, we used the confidence interval (CI) to overcome this problem. The CI is an estimate calculated from the statistics of the observed data, indicating a range of possible values for the parameter; thus, it is used to identify genes that are specifically highly expressed in particular tissues. We calculated the lower bound of the 99% confidence interval (LCI) ${{{{\rm{O}}}}}_{{{{\rm{Li}}}}}$ (${{{\rm{i}}}}$th gene’s LCI) of the genes obtained in the first step for each tissue (heart, lung and stomach) and calculated the upper bound of the 99% confidence interval (UCI) ${{{{\rm{T}}}}}_{1{{{\rm{Ui}}}}},\cdots ,{{{{\rm{T}}}}}_{42{{{\rm{Ui}}}}}$ for the remaining 42 tissues. Then, we extracted the genes that had a higher LCI for each tissue (heart, lung and stomach) than the maximum UCI among the 42 tissues $({{{{\rm{O}}}}}_{{{{\rm{Li}}}}} > \,{{{\rm{max }}}}({{{{\rm{T}}}}}_{1{{{\rm{Ui}}}}},\cdots ,{{{{\rm{T}}}}}_{42{{{\rm{Ui}}}}}))$. As a result of CI filtering, we identified candidate genes that are highly expressed in particular tissues (153 genes in heart, 189 genes in lung and 73 genes in stomach). Step 3: Although highly expressed tissue-specific genes of the three organs were previously identified, among the rest of the tissues, some genes had expression values in the top 25% of the samples that were higher than those in the tissues of interest (heart, lung, stomach) because of the large variation in the expression values. To eliminate these false-positive results, we performed quantile comparison between each of 3 tissues (heart, lung, stomach) and the remaining 42 tissues. First, we set the top 25% RPKM value of each tissue (heart, lung, stomach) as ${{{{\rm{O}}}}}_{{{{\rm{qi}}}}}$ and set the top 25% RPKM values of the remaining 42 tissues as ${{{T}}}_{1{{{\rm{qi}}}}},\cdots ,{{{T}}}_{42{{{\rm{qi}}}}}$. Then, we selected the genes that met the following conditions: ${{{O}}}_{{{{\rm{qi}}}}}\, > \, {{t}}\times {{\max }}({{{T}}}_{1{{{\rm{qi}}}}},\cdots ,{{{T}}}_{42{{{\rm{qi}}}}}),{{t}}=1.05$. Through quantile comparison, we defined the final organ-specific expressed genes (143 genes of heart, 145 genes of lung and 73 genes of stomach). To construct a gene panel that can reflect the characteristics and functions of each tissue, we added not only organ-specific expressed genes but also genes related to tissue functions. As a result, three organ-specific gene panels were constructed: a heart-specific gene expression panel with 144 genes, a lung-specific gene expression panel with 149 genes and a stomach-specific gene expression panel with 73 genes (Supplementary Dataset 1).

### Validation and characterization of Organ-GEPs

In the heatmap, the expression of each panel shows the specificity for its own tissue compared with other tissues. Moreover, in multigroup discriminant analysis (MDA), the expression panels for the lung, heart, and stomach were clearly separated from those of other tissues, suggesting that we were successful in constructing Organ-GEPs for the calculation of organ-specific similarity (Fig. 1b, c). To confirm that each Organ-GEP reflected organ-specific functions and characterizations, we performed Ingenuity Pathway analysis (IPA) using each Organ-GEP (Fig. 1d). LuGEP was associated with “inflammatory response, formation of lung, inflammation of respiratory system component, permeability of lung, fibrosis of lung and cellular infiltration of lung”. These results suggested that LuGEP reflects lung function. HtGEP was related to “contraction of heart, heart rate, contraction of cardiac muscle, cardiogenesis, organization of muscle cells, familial cardiomyopathy, muscle contraction, and enlargement of heart”. Finally, StGEP was associated with “secretion of gastric acid, morphology of gastric mucosa, gastric lesion, secretion molecule, morphology of digestive system, and peptic disorder”. Thus, to calculate the similarity between hPSC-derived organoids/cells and their respective organs, we constructed Organ-GEPs for the lung, heart and stomach, and each panel reflected the functions of each organ.

### Validation of Organ-GEP algorithms for direct comparison between target organs and organoids

**Fig. 2:**
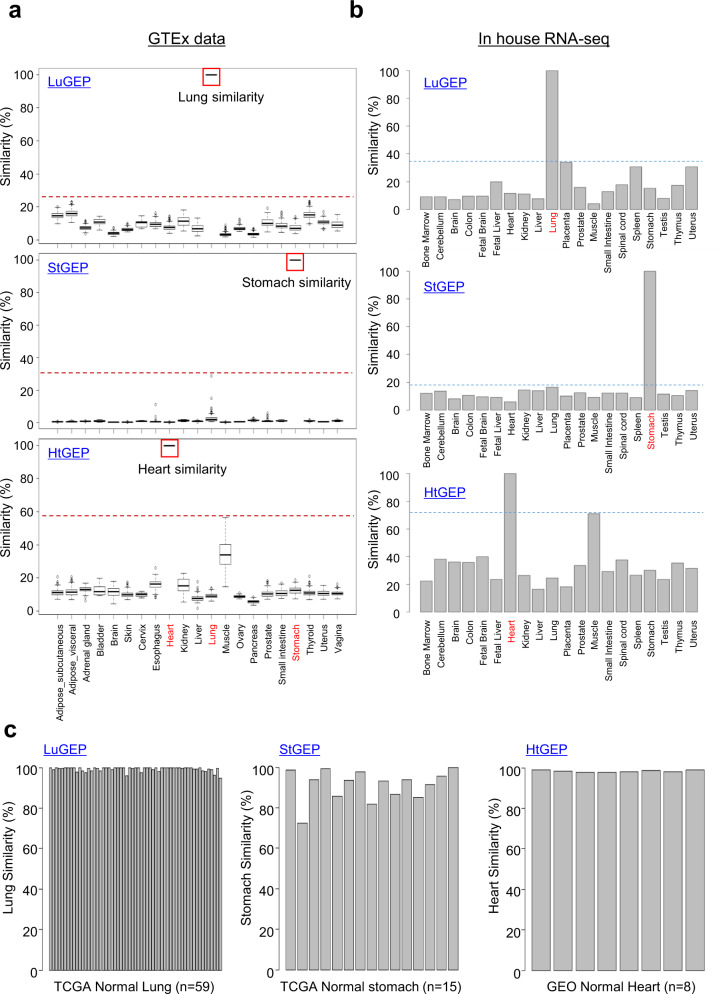
Development and evaluation of the Organ-GEP algorithm. **a** Distribution of the Organ-GEP algorithm (LuGEP, StGEP, HtGEP) scores. RNA-seq data were downloaded from the GTEx portal. A box plot shows the interquartile (IQR) range of each algorithm score in 21 tissue types. The boxes denote the lower (Q1) and upper (Q3) quartiles, and the center line is the median. The whiskers represent the highest and lowest values that are not outliers or extreme values. Circles beyond the whiskers represent outliers and extreme values. The GTEx tissues of each Organ-GEP, i.e., lungs of LuGEP, have 100% similarity scores. **b** Validation of the Organ-GEP algorithm using in-house data (20 tissue samples). Pooled RNA-seq data of each tissue were purchased from Clontech. **c** Validation of the Organ-GEP algorithm with TCGA and GEO data. The organ similarity scores of live samples for LuGEP, StGEP, and HtGEP.

We established organ-specific calculation algorithms called Organ-GEPs for determining cutoff values of organ-specific genes via calculation of the distance based on the standard gene expression vectors between organoids (see Materials and Methods). To assess the algorithm, we first used GTEx public data. In Fig. 2a, LuGEP calculated 100% similarity to lung tissue for 320 lung tissue samples (*p* > 0.001); other tissues showed <20% similarity to lung tissue, implying that the LuGEP algorithm could specifically distinguish lung tissues among all human tissues. In addition, similar to LuGEP, HtGEP presented 100% similarity to 412 heart tissue samples (*p* > 0.001), but the HtGEP score also showed high similarity to muscle. In the StGEP analysis, the similarity to the stomach was 100% (*p* > 0.001) for 193 gastric samples. The other tissue samples presented under 10% similarity as calculated by the StGEP algorithm. Thus, we confirmed the organ-specific panels and algorithm for the prediction of the target organ similarity of hPSC-derived organoids and cell types. Additionally, we produced RNA-seq data with total RNA from 20 tissues purchased from Clontech (Human Total RNA Master Panel) and calculated the similarity using organ panels and algorithms. The results showed 100% similarity to the lung, stomach, and heart (Fig. 2b). To further validate the algorithms in Organ-GEPs, we used TCGA (normal live lung and stomach) and GEO (normal heart; GSE133054) sample data to calculate organ similarity by the Organ-GEP algorithms. For the lung and stomach, 59 live lung samples had an average organ similarity of 99% (LuGEP), and 15 live stomach samples had average organ similarity of 91% (StGEP). Eight live heart samples had an average organ similarity of 98% (Fig. 2c). In summary, we suggest the a method for the quantitative calculation of similarity between human organs and hPSC-derived organoids and cells.

### LuGEP algorithm can calculate the similarity of hPSC-derived lung organoids to lung tissue

**Fig. 3:**
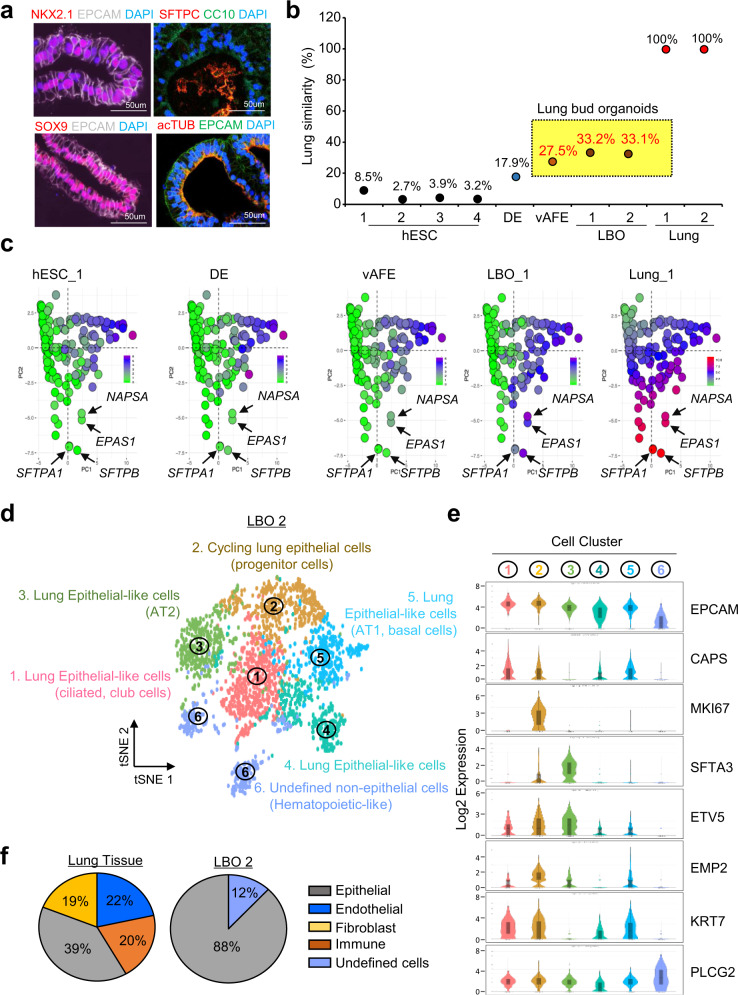
Calculation and validation of lung-specific similarity of hESC-derived LBOs. **a** Immunofluorescence staining images of hLBO (D56) sections. LBOs (*n* = 3) were stained with anti-NKX2.1, anti-SOX9, anti-SFTPC, anti-acTUB (Cy3, red), anti-EPCAM, anti-CC10 (Alexa Fluor 488, white or green) and DAPI (blue). Scale bar, 50 μm. **b** The result of the LuGEP algorithm in hESC-derived LBOs and human lung tissue. **c** GEAP analysis with LuGEP results in hESC-derived LBOs and human lung tissue. One dot indicates one gene, and differences in color indicate differences in gene expression. (green; 0, red; 10). **d** tSNE plots of the scRNA-seq data of the LBO-2 samples and cell annotation of each cell cluster. **e** Violin plots showing the single-cell distribution of lung-specific markers in each LBO-2 cell cluster. Boxes were displayed the interquartile range (IQR) from lower quartile (q1) to upper quartile (q3) with the middle dotted line and straight line representing the mean and median values, respectively; whiskers denote the highest and lowest values within 1.5 times of IQR. **f** Pie chart for the cell-type proportions in human lung tissue and LBO-2 cells.

To validate the tissue similarity of human lung organoids by LuGEP and the algorithm, we generated LBOs by a stepwise differentiation method that mimics the development process of the human lung, as previously described^26^ (see “Methods”). First, undifferentiated hESCs were differentiated into definitive endoderm (DE, d4) cells expressing the surface markers CXCR4 and C-kit. DE cells were further differentiated into ventralized anterior-foregut endoderm (vAFE, d21) and subsequently into LBOs (days 56–59) by embedding in Matrigel (Supplementary Fig. S1a). Differentiated LBOs showed branching morphologies similar to those observed in the developing lung and was composed of EPCAM-positive epithelium, which coexpressed the pulmonary endoderm marker NKX2.1 and the distal pulmonary endoderm progenitor marker SOX9. Some cells expressed pulmonary cell-type-specific markers, including alveolar type 2 cells (SFTPCs), club cells (CC10), and ciliated cells (acTUBs) (Fig. 3a). Before scoring the lung differentiation status of hESCs using the LuGEP algorithm, we verified the expression of representative stage-specific markers at each stage by quantitative RT-PCR (Supplemental Fig. S1b).

To calculate the similarity of lung organoids to lung tissue using the LuGEP algorithm, we performed LuGEP analysis on hESC, DE, vAFE, LBO, and human lung tissue samples. Figure 3b shows that hESCs and lung tissue samples exhibited 2.7%–8.5 and 100% similarity to the lung, implying that LuGEP analysis could accurately distinguish lung tissue from other cells. However, DE had 17.9% similarity, and vAFE, LBO-1, and LBO-2 had 27.5%, 33.4%, and 33.1% similarity, respectively, to the human lung (Fig. 3b). Additionally, compared to human lung tissue, each lung organoid exhibited different gene expression patterns in LuGEP. In gene expression alteration plot (GEAP) analysis, we observed that the colors of each dot were similar to those of the human lung. The expression levels of type 2 alveolar cell markers (SFTP1 and SFTPB) gradually increased depending on the day of the LBO. Endothelial PAS domain-containing protein 1 (EPAS1 or HIF-2 alpha) and NAPSA (aspartic proteinase) were gradually induced (Fig. 3c).

To further verify the lung-specific similarity of LBO in the context of cellular diversity, we performed single-cell RNA-sequencing (scRNA-seq) analysis with the same LBO-2 batch that showed 33.1% similarity in LuGEP analysis (Fig. 3b). Cell clustering and cell-type annotation with cell-type-specific markers revealed that LBO-2 consisted of seven lung epithelial-like cells (lung epithelial progenitor cells, alveolar type 2 and 1 cells, ciliated cells, basal cells, and a small number of club cells and mucose cells) and undefined nonepithelial cells (expressing some hematopoietic-related genes but not expressing the canonical immune cell marker PTPRC) (Fig. 3d, e and Supplementary Fig. S2). According to a recently reported scRNA-seq study on human lung tissue^27^, the human lung is composed of 39% epithelial cells, 22% endothelial cells, 20% immune cells, and 19% stromal cells. In contrast, LBO-2 was primarily composed of 88% lung epithelial-like cells and 12% undefined cells, but stromal and endothelial cells and well-differentiated immune cells were not detected (Fig. 3f). In addition, since we used early LBO (days 56 to 59), lung-specific epithelial cells in LBO also showed immature fetal-like characteristics, with relatively low expression of the functional lung markers SFTPB and SCGB1A1 and high expression of the embryonic lung transcription factor SOX9 (Supplementary Fig. S2). Thus, the prediction of 33% lung similarity by the LuGEP algorithm might reflect both the low cell diversity centered on parenchymal lung epithelial cells, which constitute only 39% of lung tissue, and the fetal-like immature characteristics of the differentiated cells.

### Calculation of the similarity of human antrum-like gastric organoids (GOs) to stomach tissue with the StGEP algorithm

**Fig. 4:**
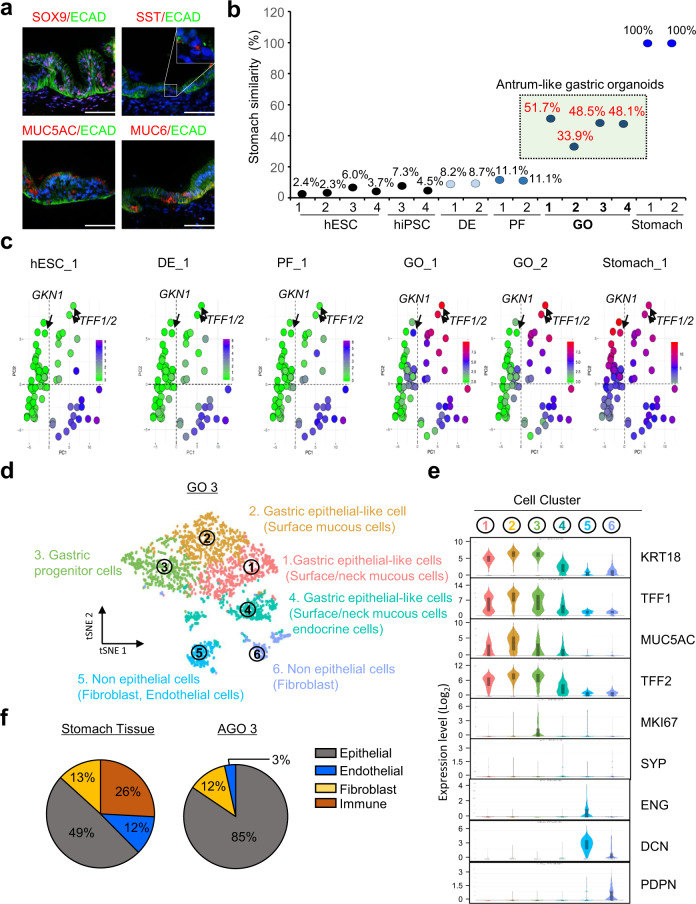
Application of StGEP analysis with hESC-derived GOs. **a** Representative immunofluorescence staining images of GO sections (*n* = 3). Anti-ECAD (Alexa Fluor 488; green), anti-SOX9, anti-SST, and anti-MUC5AC, and anti-MUC6 antibodies (Alexa Fluor 594; red) were used. DAPI (blue). Scale bar, 100 μm. **b** The result of the StGEP algorithm with hESC-derived GOs and human stomach. **c** GEAP analysis with StGEP results. The pattern of colors and dots shows the StGEP results regarding each developmental stage of hESC-derived GOs (green; 0, red; 15). **d** tSNE plots showing cell types for the GOs. **e** Violin plots showing the expression distribution of gastric marker genes in six cell clusters of GOs. Boxes were displayed the interquartile range (IQR) from lower quartile (q1) to upper quartile (q3) with the middle dotted line and straight line representing the mean and median values, respectively; whiskers denote the highest and lowest values within 1.5 times of IQR. **f** The proportion of cell types in human stomach tissue and GOs.

To analyze the tissue similarity of GOs using StGEP, we generated hESC-derived antrum-like GOs (see Materials and Methods). During stepwise GO differentiation, stage-specific markers were expressed (Fig. 4a and Supplementary Fig. S3a, b). After 30–34 days, we observed 3D-cultured single spheroids, which transformed into spherical organoids, including ones exhibiting stomach antrum-like glandular morphologies, in Matrigel (Supplementary Fig. S3c). We performed gastric similarity analysis on GOs using the StGEP algorithm. Figure 4b shows that hESCs and posterior foregut (PF) cells presented 2–6% and 11% similarity, respectively. However, GO showed 51.7%–33.9% similarity to human stomach tissue (Fig. 4b). In addition, StGEP showed different expression patterns in GOs and PF cells compared with the human stomach. In GEAP analysis, the expression of TFF1/2 and GKN1 gradually increased between PF cells and GOs. Moreover, as GO-1 and GO-2 showed 51.7% and 33.9% similarity to stomach tissue, respectively, we identified a gene expression difference between GO-1 and GO-2 (Fig. 4c). Moreover, we performed scRNA-seq analysis using the same batch of antrum GO-3, which showed 48.5% stomach-specific similarity in StGEP analysis (Fig. 4b). The tSNE plot and violin plot analysis with canonical cell-type markers revealed that the GO consisted of epithelial-like cells (surface/neck mucous-like cells, endocrine cells), nonepithelial-like cells (fibroblasts), and gastric progenitor-like cells (Fig. 4d, e and Supplementary Fig. S4). The antrum GOs did not express *ATP4A*, which is a parietal cell marker in the gastric fundus (Supplementary Fig. S4). The gastric tissue consisted of 49% epithelial cells, 26% immune cells, 13% fibroblasts, and 12% endothelial cells^28^, but the GOs mainly consisted of epithelial cell types (85%), fibroblasts (12%), and endothelial cells (3%). (Fig. 4f). These results imply that the somewhat low similarity percentage of GOs in StGEP analysis may reflect the lower cellular diversity in GOs than in human stomach tissue. Thus, using the StGEP analysis results, we quantitatively evaluated the quality of GOs by comparing each sample directly to the human stomach.

### Generation of cardiomyocytes from hESCs and calculation of similarity to heart

**Fig. 5:**
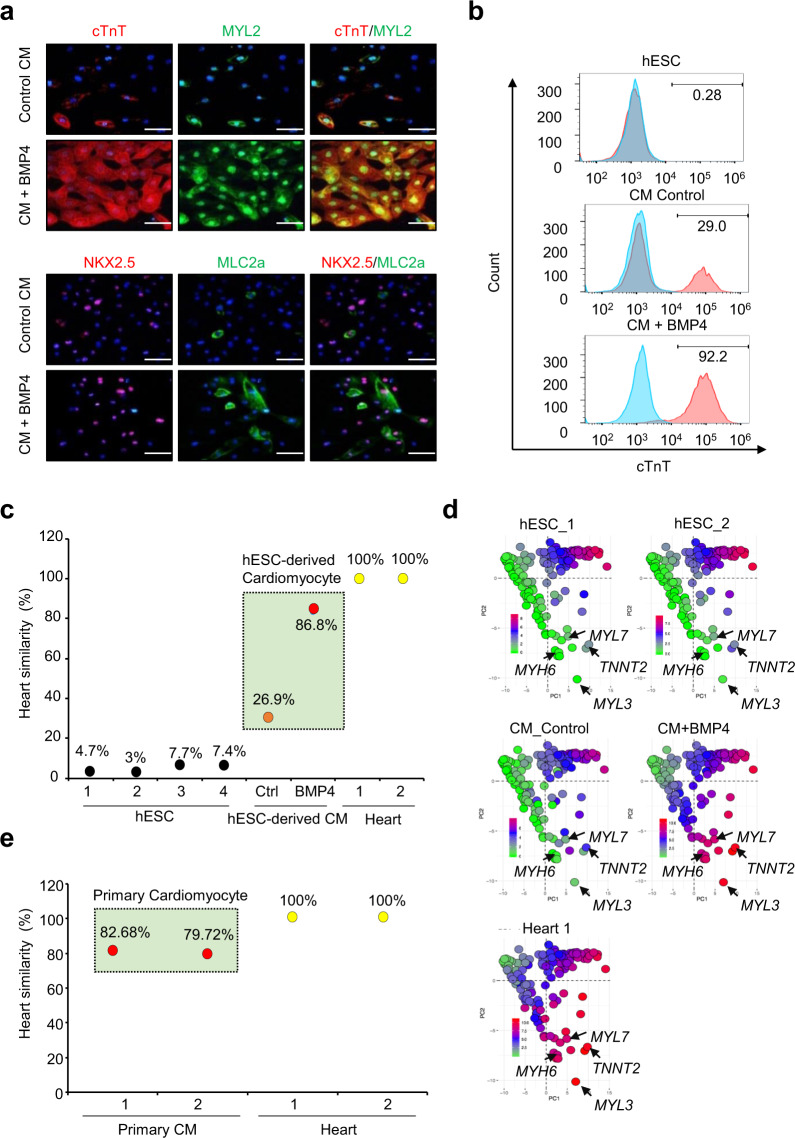
Quantitative similarity of hESC-derived CMs to heart tissue using HtGEP analysis. **a** Immunofluorescence staining images of hESC-derived CMs (*n* = 3) and CMs+BMP4 (*n* = 3). Anti-cTnT and anti-NKX2.5 antibodies (Alexa Fluor 594; red), anti-MYL2 and anti-MLC2a antibodies (Alexa Fluor 488; green), and DAPI (blue) were used. Scale bar, 50 μm. **b** FACS analysis using cardiac muscle-specific markers (cardiac troponin T, cTnT) within live cells (gated by forward and side scatter). The number indicates the differentiation rate of the hESC-derived CMs and CMs+BMP4. **c** The result of the HtGEP algorithm for hESC-derived CMs and human heart tissue. **d** GEAP analysis of the HtGEP results. Different colors indicate the gene expression levels from HtGEP in hESC-derived CMs (green; 0, red; 10). **e** The result of the HtGEP algorithm with primary CM mRNA.

Next, to verify the utilization of the HtGEP algorithm, we differentiated CMs from hESCs by modulating Wnt signaling (see Materials and Methods). The differentiated CMs with BMP4 exhibited high expression of cardiac-specific markers (Supplementary Fig. S5). We also detected high expression of cardiac transcription factors (NKX2.5), cardiac muscle markers (cTnT), ventricular CM markers (MYL2), and atrial CM markers (MLC2a) in the differentiated CMs with BMP4 compared to the control CMs by immunocytochemical analysis (Fig. 5a). Moreover, in FACS analysis of cardiac muscle-specific markers (cardiac troponin T, cTnT), hESCs and the control CMs showed 0.28 and 29% differentiated yields. However, differentiated CMs with BMP4 presented a 90% differentiated yield (Fig. 5b).

We performed HtGEP analysis on hESCs and differentiated CMs. As shown in Fig. 5c, the undifferentiated samples had 3.0–7.7% similarity, and differentiated CMs without BMP4 had 26.9% similarity. Moreover, differentiated CMs with BMP4 had 86.8% similarity to heart tissue (Fig. 5c). GEAP analysis results showed the difference in expression patterns between the control CM and CM + BMP4 groups (Fig. 5d). CMs treated with BMP4 during differentiation, expressed CM markers (MYH6, MYL3/7, TNNT2) in a pattern dramatically similar to the expression pattern of human heart tissue. Moreover, HtGEP analysis using available primary human CMs showed 82.68% and 79.72% similarity to human heart tissue, which is close to the values for differentiated CMs with BMP4 (Fig. 5e). Thus, using HtGEP analysis, we determined the heart-specific similarity of CMs and quantified the effects of BMP4 as a CM differentiation factor.

### User interface of the W-SAS

**Fig. 6:**
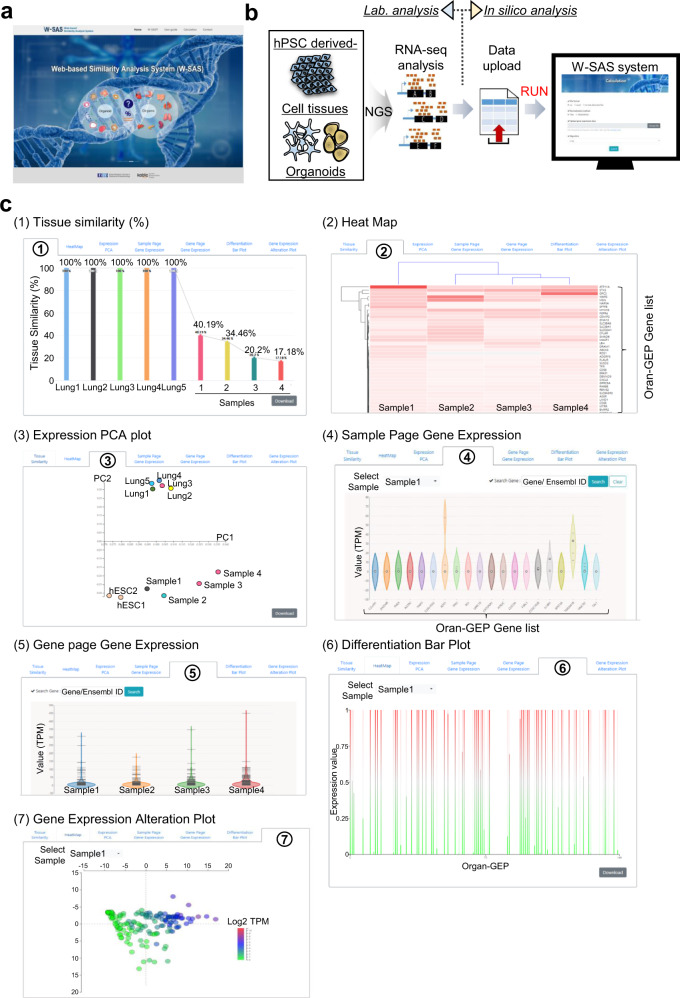
W-SAS is a user-friendly interface for the quantitative calculation system for the similarity of hPSC-derived organoids and cells to organs. **a** Main page of W-SAS. **b** A workflow for W-SAS. Using the RNA-seq results of hPSC-derived organoids and cells, W-SAS can calculate the similarity of each organoid and cell to the human target organ. **c** Results page showing similarity of the hPSC-derived organoids. The results are divided into five parts: similarity percentage (1), heat map (2), PCA plot (3), gene expression panel of Organ-GEPs or samples (4 and 5), differentiation bar plot (6), and GEAP (7).

In this study, we established a similarity calculation system for hPSC-derived lung organoids, GOs and CMs. To provide the calculation algorithm directly to researchers, we developed a user-friendly interface (W-SAS, http://kobic.re.kr/wsas) for the calculation of the organ similarity hPSC-derived organoids and cells (Fig. 6a). W-SAS is easy to use for the calculation of similarity. Figure 6b displays the RNA-seq results of each hPSC-derived organoid and cell uploaded to the W-SAS interface and the calculated and presented results showing similarity to the appropriate organ for each sample.

The W-SAS was constructed as a two-tiered server-client architecture. The server side was developed using Java Spring Framework with Java Development Kit (JDK) 1.7, and the client side was implemented using Bootstrap CSS Framework and JavaScript libraries, including Jquery, Ajax and D3.js. W-SAS is divided into two sections. The first section is the data upload. In this section, users upload the RNA-seq result (FPKM/RPKM, TPM values) formatted with Excel (csv, excel, txt files), select the algorithm for each sample, and finally submit the data for similarity calculation. In the second section, after the calculation, 7 types of results are presented. (1) Organ similarity as a percentage and a bar graph. (2) A heatmap showing the expression levels of each gene in Organ-GEP for each sample. (3) The PCA result to determine the relationship between the target organ and organoids. (4) and (5) Information suggested by W-SAS on the expression profile of each gene in each sample. (6) and (7) High- and low-expression genes in the hPSC-derived organoids and cells compared to the human target organ shown by bar plot (6) and GEAP (7) (Fig. 6c). Thus, using the W-SAS interface, researchers can rapidly receive important information about the similarity and gene expression gene patterns of hPSC-derived organoids and cells compared to human target organs. Using the information from W-SAS, researchers can develop high-quality organoids via the regulation of each panel of genes or the alteration of culture conditions to improve organ similarity.

## Discussion

We constructed a system and a user interface (W-SAS) for predicting the similarity of hPSC-derived organoids and cells to three organs based on RNA-seq analysis. After calculation of the organoid data, researchers can receive information to improve the quality of the organoids, such as percent similarity (%) and gene expression patterns, via direct comparison between the target human organs and the organoids.

The first advantage of W-SAS is a direct comparison between human target organs and differentiated organoids/cells. After the generation of hPSC-derived organoids/cells, W-SAS was used to calculate similarity to the target organ, showing a high percentage of similarity of the differentiated organoids/cells to the corresponding target organs. Second, W-SAS provides a researcher with an adequate number and names of target organ-specific genes in the target human organs and the organoids. This information can help to develop high-quality organoids via the regulation of insufficiently target organ-specific genes, such as overexpressed or depleted genes. The third advantage is the infinite expandability. For calculation of the similarity to a target organ, a target-specific gene expression panel and algorithm are needed. Here, we developed calculation algorithms and organ-specific gene-screening pipelines using RNA-seq data and applied them to all human organs to calculate the similarity to hPSC-derived organoids. Finally, W-SAS is a user-friendly interface for the calculation of similarity to a target organ from the RNA-seq data of hPSC-derived organoids. To develop W-SAS, we focused on user comfort and organ expandability and designed a simple web page for researchers (“Data uploading”-“RUN”-“Result”). Interpretation of the similarity percentage is also clearly provided to researchers. Moreover, the construction of an Organ-GEP and analytical algorithm accessed through this interface could serve researchers worldwide for the calculation of similarities to various organs.

However, W-SAS has some disadvantages. W-SAS was constructed based on transcriptome analysis using the GTEx database. Transcription data or computational network analysis are useful tools for the evaluation of lineage specifiers or differentiation stages during stem cell differentiation^29–31^. Moreover, transcriptome analysis showed higher sensitivity and reproducibility than proteome analysis. However, although transcriptome analysis can predict tissue-specific features, RNA-seq data do not reflect 100% of cell functions because the total RNA is not all translated into proteins^31^. W-SAS can analyze whether organ-specific genes are acquired at each stage of differentiation from hPSCs. Thus, in this study, organ development- and function-associated genes were manually collected by keyword searches of the PubMed database to compensate for the disadvantage of the W-SAS calculation system. In addition, for organ-specific genes in Organ-GEPs, we included organ function-associated genes that show significant temporal expression in the stomach, heart, and lung and that show core signatures enriched with functional genes. However, to verify the cell functions suggested by the similarity percentage, researchers must perform biochemical analyses to compensate for the shortcomings of transcriptome analysis.

Moreover, we addressed various issues with the W-SAS system. First is common cell types issue for Organ-GEP analysis. Since our similarity prediction mechanism is based on tissue-specific gene expression, there is a concern that gene expression from nonparenchymal cells, the common cell types across various organs, such as endothelial cells, immune cells and fibroblasts, may be ignored. However, many recent studies have reported that nonparenchymal cells in different organs have tissue-specific characteristics^32–35^. IPA analysis of LuGEP examines many parts related to the function of nonparenchymal cells, implying the involvement of lung-specific genes in nonparenchymal cells, suggesting that Organ-GEP can cover the tissue-specific heterogeneity of the common nonparenchymal cell population. Nevertheless, we do not rule out the possibility that our organ similarity prediction system does not reflect the exact cell composition of organs, since the heterogeneity of parenchymal cells and nonparenchymal cells in tissues is not at the same level,

The second category is site-specific issues in W-SAS analysis. Organoid technology has been recently developed as a method that recapitulates region-specific tissues or mimics whole organs^12,13,26,36–39^. As we developed LuGEPs, StGEPs, and HtGEPs based on whole-organ RNA-sequencing data, it is difficult to accurately predict the tissue similarity of region-specific organoids with the current version of Organ-GEP. For example, StGEP presented a total gene expression panel consisting of region-specific genes from the fundus and antrum. The fundus and antrum regions of the stomach have different functions, and cell-type-specific gene expression corresponds to distinct cell types^40^. There is a report of hPSC-derived GOs that contain either antral or fundic epithelium^41^. Actually, antrum-like GOs without parietal cells and chief cells and fundus-like GOs had 33.92–51.71% and 56.35–58.44% similarity scores, respectively (Fig. 4b and supplementary Fig. S6). Given that this analysis cannot determine the uniqueness of each functional region, a new gene expression panel reflecting region specificity in organs and a corresponding quantitative calculation system are also needed to accurately characterize the region-specific similarity of organ models.

The third issue is the difficulty of demonstrating the accuracy of the W-SAS system due to organ complexity. To address this issue, we performed scRNA-seq analysis using the same batch of lung organoids and GOs used for W-SAS. scRNA-seq analysis allowed us to assess organoid differentiation in terms of cell diversity and individual cell identity. In scRNA-seq, the cell proportion of GO was more similar to that of the corresponding tissue than LBO, and the expression of representative functional markers was also observed at a high level. In the case of LBO, although diverse cell types (seven lung epithelial-like cells) were detected in organoids, the tissue similarity of cellular proportions was low due to the high cellular diversity of the target organ: it has been reported that there are 58 cell populations in the human lung^27^. LBO differentiation is restricted to epithelial cells, which are not detected among in nonparenchymal cells, but GOs contain 12% fibroblasts and 3% endothelial-like cells. In the Organ-GEP analysis, GO (48.5%) predicted a higher tissue similarity than LBO (33.1%). Thus, although these results do not accurately represent the relationship between the results of W-SAS and scRNA-seq, using scRNA-seq analysis, we could (1) validate the quality of W-SAS and (2) suggest that the high similarity percentage indicates comparable cell diversity between hPSC-derived organoids and their target organs.

In conclusion, to generate high-quality organoids from hPSCs, researchers can visit the W-SAS interface to assess the organ similarity of their own hPSC-derived organoids and receive information (similarity, heat map, PCA plot, expression patterns of panel) that can help generate high-quality organoids by engineering gene expression. Thus, feedback between W-SAS and researchers can become a vital troubleshooting step in the organoid research field.

## Methods

### Cell culture

Human PSCs were cultured through a previously reported method^23,42^. H9-hESCs (human, WiCell WA09) were purchased from the WiCell Research Institute (Madison, WI, USA), and hiPSCs were reprogrammed using episomal vectors from fibroblasts (CRL2097, ATCC, Manassas, VA)^23^. The hPSCs were maintained in hPSC medium including Dulbecco’s modified Eagle’s medium (DMEM)/F-12 (Invitrogen, Carlsbad, CA, USA), 20% knockout serum replacement (Thermo Fisher), 1% penicillin/streptomycin (Invitrogen), 1% GlutaMAX (Invitrogen), 0.1 mM β-mercaptoethanol (Invitrogen), 1% nonessential amino acids (NEAA, Invitrogen) and 8 ng/ml basic fibroblast growth factor (bFGF, R&D Systems, Minneapolis, MN, USA). The cells were passaged every 7 days.

### Transcriptome data acquisition and data preprocessing

We developed Organ-GEP to reflect the characteristics and functions of each organ to develop a model that quantitatively predicts the differentiation of heart, lung, and stomach tissues. A total of 8555 RNA-seq datasets (transcript RPKM of GTEx version 6) from 53 tissues were obtained through the publicly available GTEx database to select genes that reflected the characteristics and functions of each tissue. While the RNA-seq data on the lung and stomach provided only one RPKM dataset each, the heart is divided into two subtissues (atrial appendage and left ventricle). We used both heart subtissues as a single heart sample, and all other subtissues of the remaining tissues were used as independent data. For the classification of 52 tissues, the MDS plot was calculated with 56,238 RPKM values of all genes, and the testis was excluded because it was clearly separated from other tissues. Genes specifically expressed in the testis can lead to false-positive results when identifying organ-specific genes in three tissues. In addition, sex-specific tissues such as the ovary, uterus, vagina, fallopian tube and cervix were excluded. We also excluded whole blood and blood cell tissues because they included peripheral blood leukocytes. Finally, we prepared a total of 43 tissues (7579 samples) to construct a panel of genes to reflect the characteristics and function of the heart, lung and stomach, excluding 7 sex-specific tissues and 2 blood tissues. Data preprocessing was performed before extracting the tissue-specific expression genes. In the data preprocessing, the genes were limited to protein-coding genes, and nonexpressed genes and/or genes with extremely low-expression were filtered out. The gene sets of 43 tissues were matched to compare the gene expression differences between tissues. First, to identify genes that could play a major role in tissue differentiation, 18,818 protein-coding genes were extracted from the Ensembl gene ID information provided by the GTEx RNA-seq data. Then, we removed 1343 genes whose maximum value was <1 or whose third quantile value was <0 from all samples of the 43 tissues. Genes whose expression values were rarely measured in all tissues were not of interest, and by eliminating genes with low expression values, we can estimate the mean-variation relationship of data with high statistical reliability. Finally, we transformed the expression values into the log2 scale to normalize a total of 17,475 gene sets.

### Construction of the analytical algorithm

To construct an analytical algorithm, we used standard gene expression vectors that can distinguish between organs and undifferentiated organoids by using the expression values of organ-specific gene expression panels that reflect the characteristics and functions of each tissue. The standard gene expression vectors were defined in terms of the upper boundary of the organ (minimum expression value of the organ) and the lower boundary of the undifferentiated organoid (minimum expression value of the undifferentiated organoid). Based on the score boundaries, the status of differentiation was measured quantitatively by calculating the distance between the organ and the undifferentiated tissues. Using 73 heart samples and two undifferentiated organoid samples, we calculated the standard gene expression vector that determines the degree of tissue development. For this calculation, we performed the following steps. First, each data point was normalized to log2 (TPM + 1) values. For n genes, ${{n}}=144$ for HtGEP, the expression value of the differentiated organoid sample is $X=\left({x}_{1},\cdots ,{x}_{n}\right)$, the expression value of ${{{\rm{j}}}}$th undifferentiated organoid sample is ${U}_{j}=({u}_{{{{\mathrm{j1}}}}},\cdots ,{u}_{{{{{\mathrm{jn}}}}}})$, ${{j}}=1,2$ and the ${{{\rm{k}}}}$th expression value of the heart organ sample is ${H}_{k}=\left({h}_{k1},\cdots ,{h}_{{kn}}\right)$, ${{k}}=1,\cdots ,{{K}}$, ${{K}}=73$. To determine the standard gene expression vectors, we extracted the minimum expression values of the two undifferentiated organoid samples as ${U}_{m}=({{{\rm{min }}}}({u}_{11},{u}_{21}),\cdots ,\,{{{\rm{min }}}}({u}_{1n},{u}_{2n}))=({u}_{m1},\cdots ,{u}_{mn})$. Similarly, the minimum expression values for each gene of the heart samples were ${H}_{m}=\left({{{\rm{min }}}}({h}_{11},\cdots ,{h}_{K1}),\cdots ,{{{\rm{min }}}}({h}_{1n},\cdots ,{h}_{{Kn}})\right)=\left({h}_{m1},\cdots ,{h}_{{mn}}\right).$ ${U}_{m}$ is the minimum boundary of the undifferentiated organoid, and ${H}_{m}$ is the minimum expression boundary of the heart organ. Then, we compared the differentiated organoid *X* with *U*_*m*_ and *H*_*m*_ for each gene and set the median value of ${X}_{m}=\left({{{\rm{median}}}}\left({{u}_{m1},x}_{1},{h}_{m1}\right),\cdots {{{\rm{median}}}}\left({u}_{{mn}},{x}_{n},{h}_{{mn}}\right)\right)$ as the expression value for the differentiated organoid. This strategy was employed to adjust the expression boundary of differentiated organoids to the score boundaries; in other words, the expression value of the differentiated organoids was replaced with the standard gene expression vector when it passed the boundaries. Using these parameters, we estimated the status of differentiation by calculating the Manhattan distance among organs and undifferentiated and differentiated organoids. For quantitative assessment, we found that the score of the unknown sample approached 100 when the distance of the differentiated organoid was similar to that of the organ and approached zero if the distance was closer to the undifferentiated organoid. If ${X}_{m}={H}_{m}$, the score value is set to 100%, and the closer the ${U}_{m}$ is, the more the score decreases and the less similar the organoid is to heart tissue. The score is as follows:

$${{{{\rm{Score}}}}}_{{{{\rm{H}}}}}=\frac{{\Vert {{{{{{\mathrm{U}}}}}}}_{{{{\rm{m}}}}}-{{{{{{\mathrm{X}}}}}}}_{{{{\rm{m}}}}}\Vert }_{1}}{{\Vert {{{{{{\mathrm{U}}}}}}}_{{{{\rm{m}}}}}-{{{{{{\mathrm{H}}}}}}}_{{{{\rm{m}}}}}\Vert }_{1}}\times 100( \% )$$

$${{{\rm{where}}}}$$ $${\Vert {{{{{{\mathrm{U}}}}}}}_{{{{\rm{m}}}}}-{{{{{{\mathrm{X}}}}}}}_{{{{\rm{m}}}}}\Vert }_{1}={\sum }_{{{i}}=1}^{{{n}}}|{{{{{{\mathrm{u}}}}}}}_{{{{\rm{mi}}}}}-{{{{{{\mathrm{x}}}}}}}_{{{{\rm{mi}}}}}|$$.

The same expression value was used for the lung and stomach. For the lung, the RPKM value of the ${{{\rm{k}}}}$th lung organ sample is ${{{L}}}_{{{{\rm{k}}}}}=\left({{{l}}}_{{{{\rm{k}}}}1},\cdots ,{{{l}}}_{{{{\rm{kn}}}}}\right)$, ${{k}}=1,\cdots ,{{K}}$, ${{K}}=8$ and ${{{X}}}_{{{{\rm{m}}}}}=\left({{\min }}\left({{{x}}}_{1},{{{l}}}_{{{{\rm{m}}}}1}\right),\cdots {{\min }}\left({{{x}}}_{{{{\rm{n}}}}},{{{l}}}_{{{{\rm{mn}}}}}\right)\right)$. The score is as follows:

$${{{{\rm{Score}}}}}_{{{{\rm{L}}}}}=\frac{{\Vert {{{{\rm{U}}}}}_{{{{\rm{m}}}}}-{{{{\rm{X}}}}}_{{{{\rm{m}}}}}\Vert }_{1}}{{\Vert {{{{\rm{U}}}}}_{{{{\rm{m}}}}}-{{{{\rm{L}}}}}_{{{{\rm{m}}}}}\Vert }_{1}}\times 100( \% )$$

For the stomach, the RPKM value of the ${{{\rm{k}}}}$th stomach sample is $\,{{{S}}}_{{{{\rm{k}}}}}=\left({{{s}}}_{{{{\rm{k}}}}1},\cdots ,{{{s}}}_{{{{\rm{kn}}}}}\right)$, ${{k}}=1,\cdots ,{{K}}$, ${{K}}=21$ and ${{{X}}}_{{{{\rm{m}}}}}=\left({{\min }}\left({{{x}}}_{1},{{{s}}}_{{{{\rm{m}}}}1}\right),\cdots {{\min }}\left({{{x}}}_{{{{\rm{n}}}}},{{{s}}}_{{{{\rm{mn}}}}}\right)\right)$. The score is as follows:

$${{{{\rm{Score}}}}}_{{{{\rm{S}}}}}=\frac{{\Vert {{{{\rm{U}}}}}_{{{{\rm{m}}}}}-{{{{\rm{X}}}}}_{{{{\rm{m}}}}}\Vert }_{1}}{{\Vert {{{{\rm{U}}}}}_{{{{\rm{m}}}}}-{{{{\rm{S}}}}}_{{{{\rm{m}}}}}\Vert }_{1}}\times 100( \% )$$

### Differentiation of LBOs from hESCs

LBOs were differentiated from hESCs (H9) as previously described^26^. Briefly, hESCs were maintained on feeder cells with PSC culture medium: DMEM/F12 supplemented with 20% knockout serum replacement (Gibco), 1% GlutaMAX (Gibco), 1% NEAA (Gibco), 1% penicillin-streptomycin (Gibco), 0.1% beta-mercaptoethanol (Gibco) and 20 ng/ml FGF-basic (R&D Systems). The day before differentiation into definitive endoderm (DE) cells, hESCs were dissociated into single cells using Accutase (Millipore) and formed embryonic bodies (EBs) with serum-free differentiation medium (SFM) supplemented with 3 ng/ml BMP4 (R&D Systems) and 10 mM Y27632 (Stem Cell Technologies). The next day, the EBs were differentiated into DEs for 3 days with endoderm induction media: SFM supplemented with 100 ng/ml Activin A (R&D Systems), 2.5 ng/ml FGF-basic, 0.5 ng/ml BMP4 and 10 mM Y27632 under hypoxic conditions (5% O_2_). For patterning the anterior foregut, cells were dissociated, seeded on fibronectin-coated plates and cultured in SFM supplemented with 200 ng/ml Noggin (R&D Systems) and 10 µM SB431542 (Tocris) for 24 h. Subsequently, the medium was replaced with SFM supplemented with 1 µM IWP2 (R&D Systems) and 10 µM SB431542 for another 24 h. For further ventral patterning of the anterior foregut, the cells were cultured in ventralizing/branching medium: SFM supplemented with 50 nM all-trans retinoic acid (Sigma-Aldrich), 3 µM CHIR99021 (Tocris), 10 ng/ml BMP4, 10 ng/ml FGF10 (R&D Systems) and 10 ng/ml FGF7 (R&D Systems). After 48 h, the cells were detached by pipetting and transferred to ultralow-attachment six-well plates (Corning) to form spheroids and cultured until 21–25 days after differentiation. Ventralized anterior-foregut spheroids were embedded on Matrigel droplets for branching morphogenesis of the lung bud organoids and continued culture with ventralizing/branching media. In this study, D56 LBO was used for the analysis of lung similarity.

### Generation of human GOs

Human antrum-like GOs and fundic GOs were differentiated as described previously^13,36^. hESCs were seeded onto Matrigel-coated dishes and cultured in mTeSR1 (Stemcell Technologies, Vancouver, Canada) media. After 3 days, the cells were cultured in DE differentiation media containing RPMI-1640 and 100 ng/ml Activin A (R&D Systems) and gradually increasing FBS (Invitrogen) concentrations (0–2%) for 3 days. BMP4 (50 ng/ml, R&D Systems) was added to DE medium on the first day only. For differentiation into the posterior foregut, differentiated cells were cultured in RPMI-1640 medium supplemented with 2% FBS (Invitrogen), 2 μM CHIR99021 (Torcris, Ballwin, MO, USA), 500 ng/ml FGF4 (Peprotech, Rocky Hill, NJ, USA), and 200 ng/ml Noggin (R&D Systems). On the last day, 2 μM retinoic acid (Stemgent, Houston, Texas, USA) was added to the posterior foregut media. The 3D spheroids were collected and embedded in the Matrigel dome. After Matrigel polymerization, embedded spheroids were cultured with GO medium, including advanced DMEM/F-12 (Invitrogen), 1x N2 (Invitrogen), 1x B27 (Invitrogen), 1% penicillin/streptomycin (Invitrogen), 15 mM HEPES (Invitrogen), 2 mM l-glutamine (Invitrogen), and 100 ng/ml EGF (R&D Systems). In addition, Noggin and retinoic acid were added for the first 3 days.

For fundic GO differentiation, the posterior foregut spheroids were embedded and incubated with fundic organoid differentiation medium based on GO medium. The spheroids were incubated with maintenance medium for 14 days, including 2 μM CHIR99021 and 100 ng/ml EGF. For gastric specification, 100 ng/ml Noggin and 2 μM RA were added for the first 3 days. For morphogenesis of fundic GOs, the cells were changed to medium supplemented with EGF, CHIR99021, and 50 ng/ml FGF10 (Peprotech) for 10 days. For the differentiation of fundic stomach cell types, the fundic GOs were exposed to 10 ng/ml EGF, 50 ng/ml FGF10, 2 μM PD0325901 (Sigma-Aldrich), and 50 ng/ml BMP4 (Peprotech) for 4 days. PD0325901 and BMP4 were removed from the medium during the last 2 days. The cells were re-embedded in fresh Matrigel at 20 days. Human stomach mRNAs were purchased from Clontech and used as a positive control (San Francisco, CA, USA).

### CM differentiation

For differentiation into CMs, hESCs were seeded into 1% Geltrex (Thermo Fisher Scientific, Waltham, MA, USA)-coated tissue culture plates and grown to 70% cell confluency. For mesodermal induction, 6 μM CHIR99021 (Tocris, Bristol, UK) was treated for 2 days in CM differentiation medium consisting of RPMI-1640, 212 μg/ml l-ascorbic acid (Sigma-Aldrich, St. Louis, MO, USA), and 500 μg/ml recombinant human albumin (Sigma-Aldrich). After mesodermal induction, 2 μM Wnt-C59 was administered for another 2 days. The differentiated CMs were maintained in RPMI-1640 supplemented with 212 μg/ml l-ascorbic acid and 1x B27 supplement (Thermo Fisher Scientific). The antibodies used in this study are listed in Supplementary Table S1. Human Cardiomyocyte Total RNA (Cat No. 36044-15RNA) was purchased from CELPROGEN.

### Quantitative real-time RT-PCR (qPCR)

Total RNA was obtained from harvested cells using an RNeasy Kit (Qiagen, Hilden, Germany), and a Superscript IV First-Strand Synthesis System Kit (Invitrogen, Carlsbad, CA) was used for reverse transcription. qPCR was performed on a 7500 Fast Real-Time PCR system (Applied Biosystems, Foster City, CA, USA). Human heart RNA was purchased and used as a positive control. The sequences of the primers used in this study are presented in Supplementary Table S2.

### Immunofluorescence

Cultured cells were fixed with 4% paraformaldehyde (PFA, Sigma-Aldrich, St. Louis, MO, USA) for 15 min at room temperature (RT) and permeabilized with 0.1% Triton X-100 in PBS. After the samples were blocked with 4% bovine serum albumin (BSA), primary antibodies were incubated at 4 °C overnight. The cells were washed and incubated with secondary antibodies at RT for 1 h. DAPI (1 mg/ml; Thermo Fisher Scientific) was used to stain nuclei^43^. Images were captured with fluorescence microscopy (IX51; Olympus, Tokyo, Japan).

### Flow cytometry (FACS)

FACS analysis was performed to verify the differentiation efficiency of DE and CMs. The differentiated DE was dissociated into single cells and incubated with antibody in DPBS containing 2% FBS and 2 mM EDTA at RT for 30 min. After the cells were washed with dPBS, they were analyzed with Accuri C6 flow cytometry (BD Biosciences). The differentiated CMs were dissociated to obtain single cells, which were fixed and permeabilized using the Transcription Factor Buffer Set (BD Biosciences, San Jose, CA) according to the manufacturer’s instructions. Antibodies were diluted 1:40 and incubated at 4 °C for 40 min. After incubation, the samples were washed twice with Perm/Wash Buffer (BD Biosciences). FACS analysis was performed with an Accuri C6 flow cytometer (BD Biosciences), and the data were analyzed using FlowJo V10 software (TreeStar, USA). The antibodies used in this study are listed in Supplementary Table S1.

### Single-cell RNA-sequencing and data analysis

The organoids were chopped into ~100 × 100 μm pieces, which were incubated with the Neural Tissue Dissociation Kit (Miltenyi Biotec, NRW, Germany). First, the fragments were treated with Enzyme mix I. After 10 min, the fragments were incubated with Enzyme mix II for 20 min. Single cells from organoids were washed with 0.04% BSA and resuspended. The viable cells were qualified using Countess™ II (Thermo Fisher). Library construction was performed using 10x Chromium Single-Cell 3’ Reagent Kit v3.1 (10x Genomics, Pleasanton, CA). Samples were sequenced on the Illumina NovaSeq 6000 platform (Illumina, San Diego, CA), and preliminary sequencing results were converted to FASTQ files with 10x Cell Ranger (10X Genomics, CA). We followed the 10x Genomics standard seq protocol by trimming the barcode and unique molecular identifier (UMI) end to 26 bp and the mRNA end to 98 bp. Then, the FASTQ files were aligned to the human reference genome (GRCh38). Subsequently, we applied 10x Cell Ranger for preliminary data analysis and generated a file that contained a barcode table, a gene table and a gene expression matrix. We used Loupe Cell Browser 5.0 (10x Genomics) based on Cell Ranger (10x Genomics) for the QC, analysis, and exploration of single-cell RNA-seq data. Single cells were screened using the following criteria: >8 unique molecular identifiers (UMIs) per barcode (Log_2_), >10 expressed Log_2_ features per barcode, and <10% mitochondrial UMIs per barcode. A total of 2444 cells/LBOs and 2572 cells/antrum GOs were used for the analysis of scRNA-seq data^44^. Before dimensionality reduction, we performed principal component analysis (PCA) and then applied t-distributed stochastic neighbor embedding (tSNE) with ten principal components (PCs) for visualization. Violin plots were generated by Loupe Cell Browser v5.0 with typical tissue-specific markers.

### Reporting summary

Further information on research design is available in the Nature Research Reporting Summary linked to this article.

## Supplementary information

Supplementary information

Description of Additional Supplementary Files

Supplementary dataset 1

Reporting Summary

## Data Availability

The data discussed in this publication have been deposited in NCBI’s Gene Expression Omnibus (GEO) and are accessible through GEO Series accession number “GSE178858”. All other relevant data supporting the key findings of this study are available within the article and its Supplementary Information files or from the corresponding author upon reasonable request. Source data are provided with this paper.

## Source data

Source Data

## Code availability

Code to reproduce the analyses described in this manuscript is provided in a Zenodo repository with the identifier (10.5281/zenodo.5010881). Web-based Similarity Analytics System (W-SAS; https://www.kobic.re.kr/wsas/).
