# Supplementary material for: Development of a quantitative prediction algorithm for target organ-specific similarity of human pluripotent stem cell-derived organoids and cells
Source: Nat Commun. 2021 Jul 23;12:4492. doi: 10.1038/s41467-021-24746-w (PMC8302568; doi:10.1038/s41467-021-24746-w)
Supplement: Supplementary file 1 — Supplementary information [file 41467_2021_24746_MOESM1_ESM.pdf]

Development of a quantitative prediction algorithm for target organ-specific similarity of human pluripotent stem cell-derived organoids and cells

Mi-Ok Lee[1, 2†], Su-gi Lee[1, 2†], Cho-Rok Jung[1, 2†], Ye Seul Son[1, 2†], Jae-Woon Ryu[1], Kwang Bo Jung[1, 2], Jun-Ho Ahn[3], Jung-Hwa Oh[3], Hyang-Ae Lee[3], Jung Hwa Lim[1], Janghwan Kim[1.2], Insu Jang[1], Jinhyuk Choi[1], Jaeeun Jung[1], Kunhyang Park[1], Byungwook Lee[1], Dae-Soo Kim [1, 2], Mi-Young Son[1,2], Hyun-Soo Cho[1, 2]

[1]Korea Research Institute of Bioscience and Biotechnology, Daejeon, 34141, Republic of Korea; [2]Korea University of Science and Technology, Daejeon, 34113, Republic of Korea; [3]Korea Institute of Toxicology (KIT), Daejeon, 34114, Republic of Korea

The fie contains

Supplementary figures S1~S6

Supplementary table S1~S2

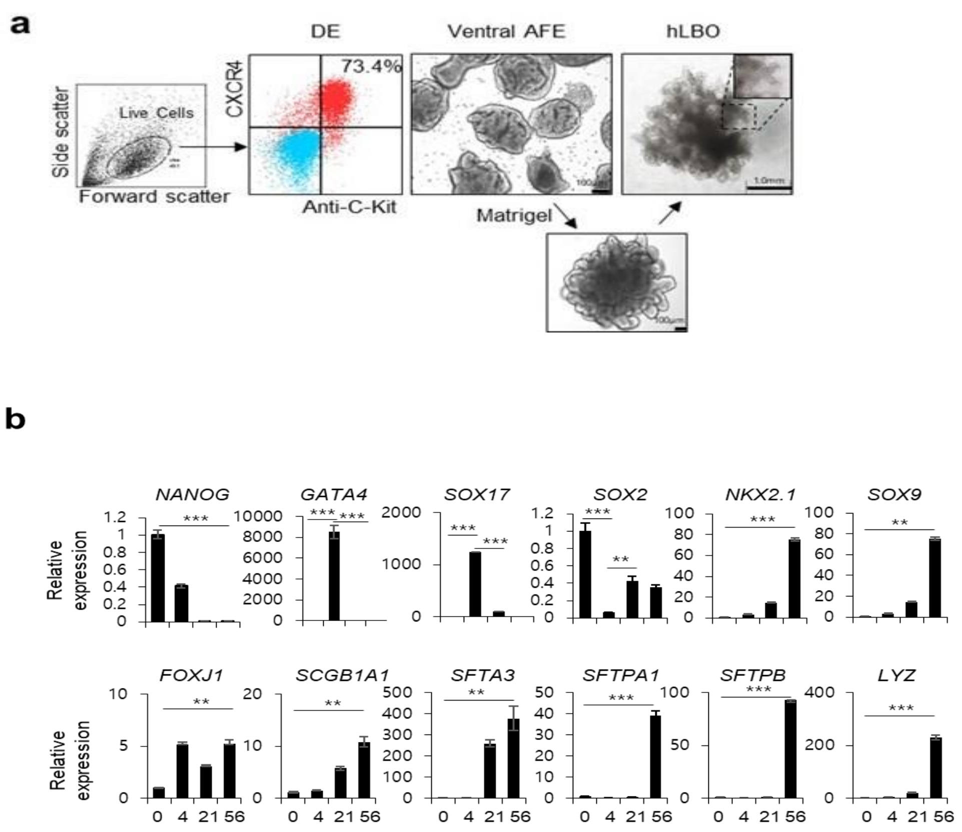

Supplementary Fig.S1 Development of hPSC-derived lung organoids. a Representative flow cytometry plot and bright field images of stepwise differentiation to LBO. Flow cytometry plot (left panel) presented the percentage of DE differentiated cells (C-Kit$^+$ CXCR4$^+$) within live cells (gated by forward and side scatter) of differentiation day 4. Blue dots indicate analysis in undifferentiated hESCs (D0), and red dots indicate FACS analysis of DE cells (D4). The number indicates the percentage of C-Kit$^+$ CXCR4$^+$ differentiated cells in DE cells (D4.) Bright field images displayed the ventralized AFE spheroids (middle panel, D21), budding spheroids after embedding in Matrigel (lower panel, D23), and branching LBO (right panel, D56 after differentiation). Scale bar, 100 μm (Ventral VEF, matrigel embedding), 1.0mm (hLBO). **b** qRT-PCR analysis of cell type-specific markers was performed in hESCs (D0), DEs (D4), vAFEs (D21) and LBOs (D56). Data represents means ± SE (n=3). The *p*-values were calculated by Student's two-tailed *t*-tests (unpaired). ** $p<0.01$, ***$p<0.001$.

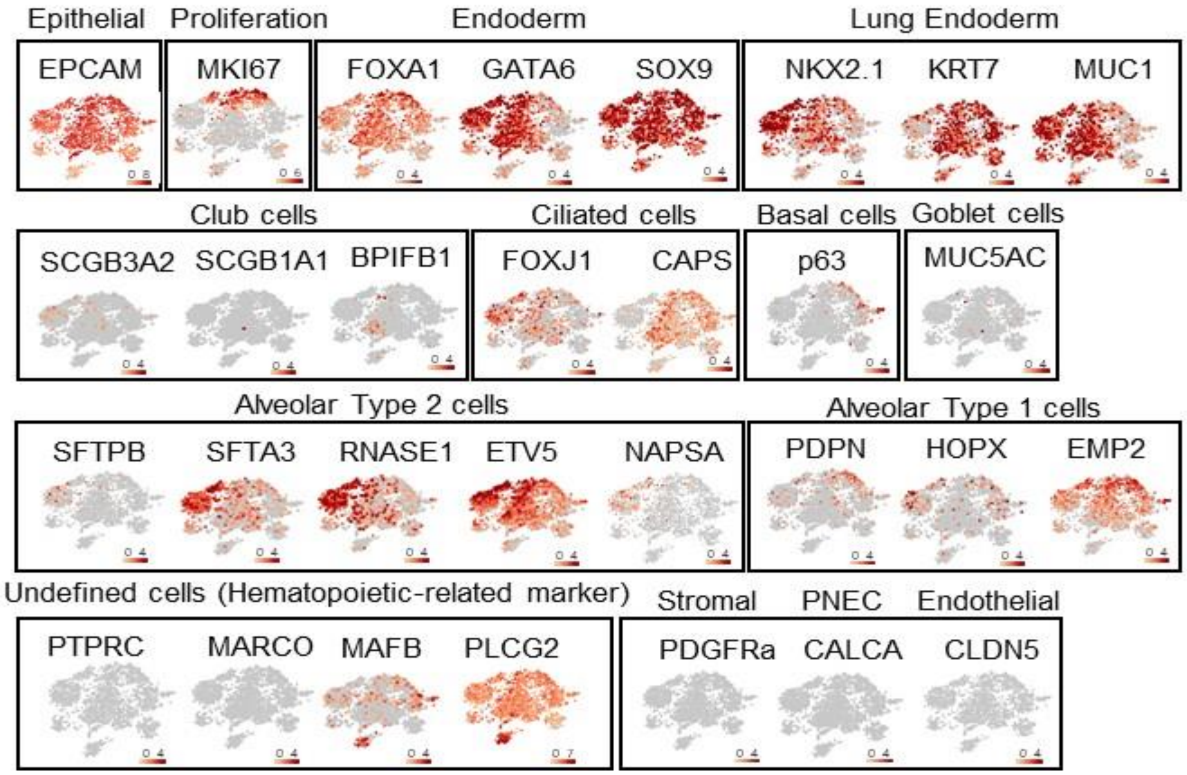

Supplementary Fig.S2 Single cell RNA-seq analysis of hPSC derived lung organoids. tSNE plots presenting the expression level of marker genes.

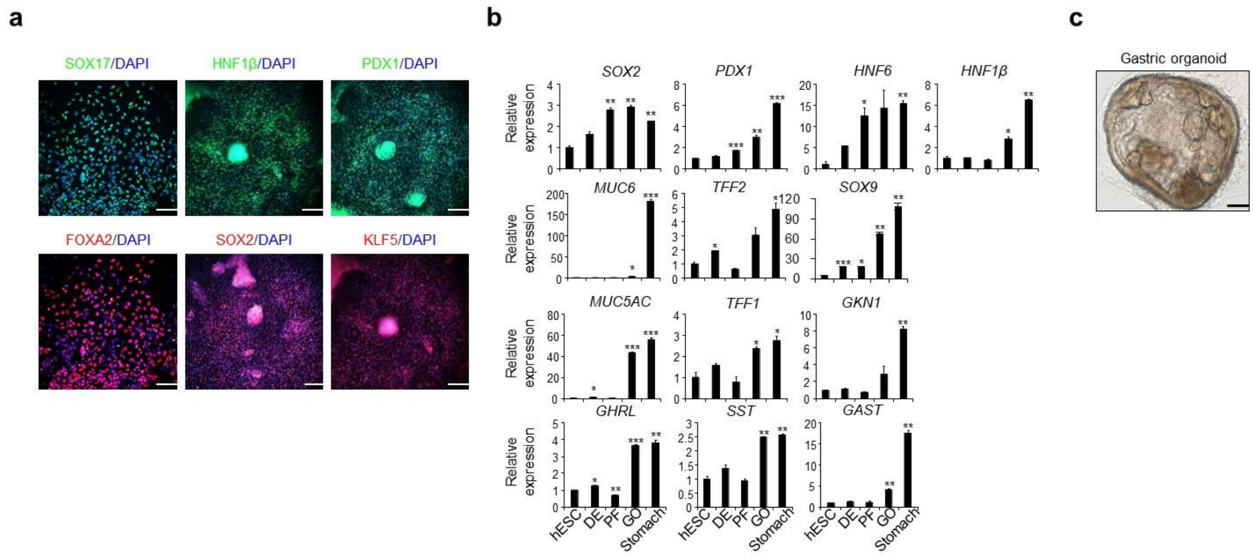

Supplementary Fig.S3 Characterization of hPSC derived GO. a Representative immunofluorescence staining images of GO sections (n=3). Anti-SOX17, anti-HNF1β, anti-PDX1 (Alexa Fluor 488; green), anti-FOXA2, anti-SOX2, and anti-KLF5 antibodies (Alexa Fluor 594; red) were used. DAPI (blue). Scale bar, 100 μm. **b** qRT-PCR analysis using cell type-specific markers in the developmental stage of hESC-derived organoids. Data represents means ± SE (n=3). The *p*-values were calculated by Student's two-tailed *t*-tests (unpaired). * p<0.05, ** p<0.01, ***p<0.001. **c** Bright field image of gastric organoids. Three independent experiments were performed. Scale bar, 100 μm.

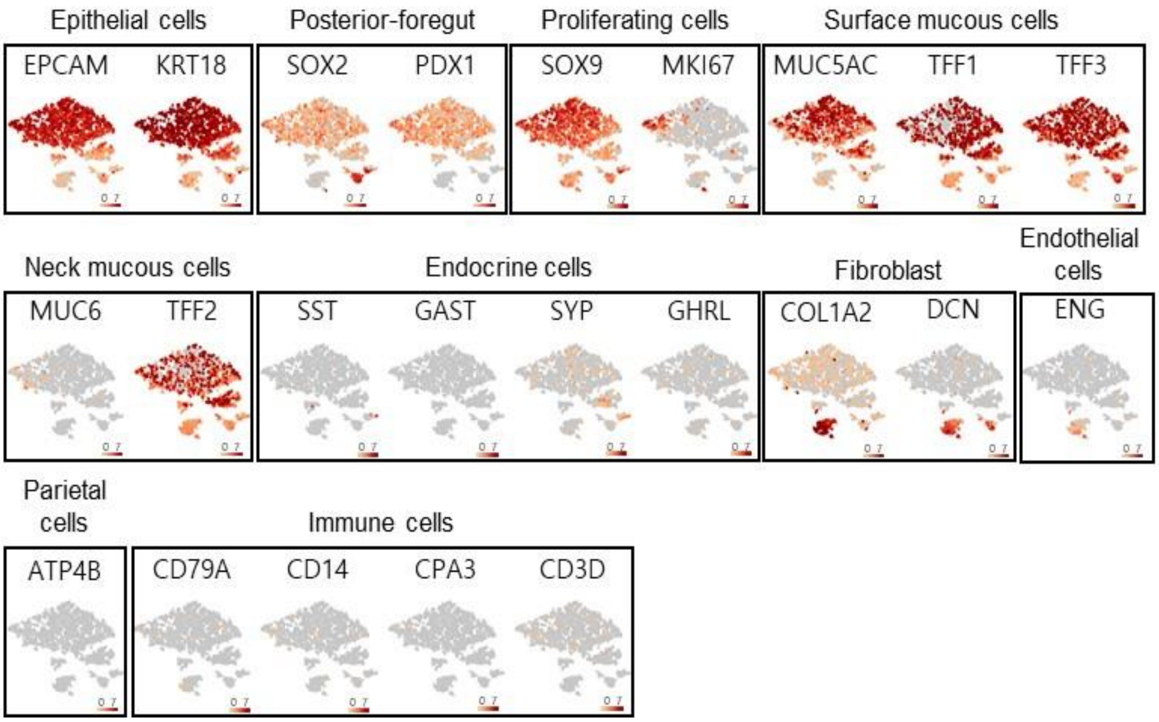

Supplementary Fig.S4 Single cell RNA-seq analysis of hPSC derived GO. tSNE plots presenting the expression level of marker genes.

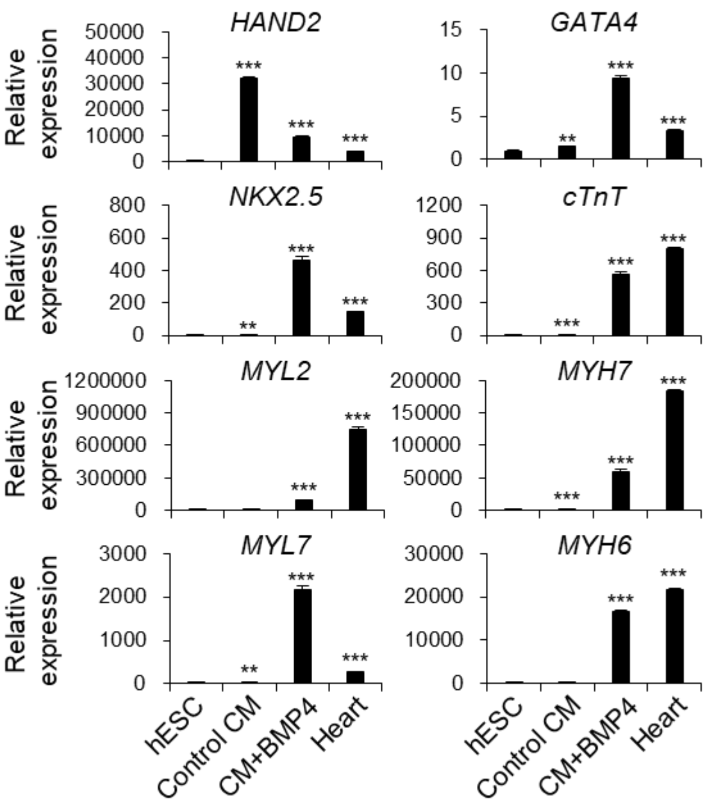

Supplementary Fig.S5 Characterization of hPSC-derived CMs. qRT-PCR analysis using cardiac transcription, muscle, ventricular, and atrial markers in the developmental stage of hESC-derived CMs. Data represents means ± SE (n=3). The *p*-values were calculated by Student's two-tailed *t*-tests (unpaired). * p<0.05, ** p<0.01, ***p<0.001.

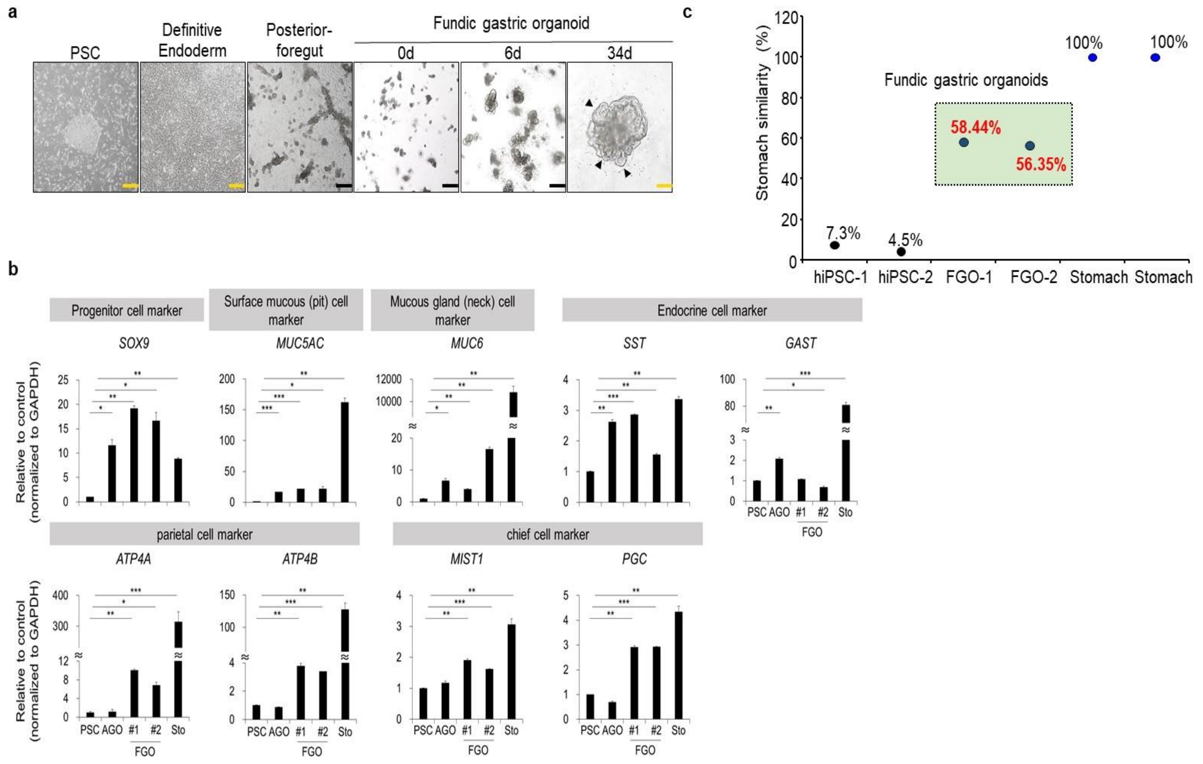

Supplementary Fig.S6 StGEP analysis with hPSC-derived fundic gastric organoids. a Bright field image of fundic GO (n=3). Black Scale bar, 200 μm, Yellow scale bar, 500 μm. **b** qRT-PCR analysis using cell type-specific markers in the developmental stage of hiPSC-derived gastric organoids. Data represents means ± SE (n=3). The *p*-values were calculated by Student's two-tailed *t*-tests (unpaired). * p<0.05, ** p<0.01, ***p<0.001. **c** The result of the StGEP algorithm with hiPSC-derived fundic GO and human stomach.

Supplementary Table S1. List of antibodies used in this study

Antibodies	Catalog No.	Company	Dilution
Gastric organoid markers			
anti-FOXA2	07-633	Millipore	1:100
anti-SOX17	MAB1924	R&D system	1:50
anti-HNF1β	sc-7411	SantaCruz	1:100
anti-PDX1	AF2419	R&D system	1:50
anti-SOX2	MAB2018	millipore	1:100
anti-KLF5	ab137676	abcam	1:100
anti-SOX9	sc-20095	SantaCruz	1:50
anti-SST	A0566	Dako	1:200
anti-MUC5AC	ab78660	Abcam	1:200
anti-MUC6	sc-33668	SantaCruz	1:50
anti-ECAD	610182	BD biosciences	1:200
anti-ECAD	AF648	R&D system	1:200
Cardiomyocyte markers			
anti-cTnT	Ab64623	abcam	1:100
anti-MYL2	10906-1-AP	Proteintech	1:50
anti-NKX2.5	AF2444	R&D	1:100
anti-MLC2a	311011	Synoptic system	1:100
anti-cTnT-PE	564767	BD	1:40
anti-Mouse IgG1-PE	130-092-212	Miltenyi Biotec	1:40
Lung bud organoid markers			
anti-NKX2.1	Ab76013	Abcam	1:200
anti-SOX9	AB5535	Millipore	1:200
anti-EPCAM	2626	Cell Signaling	1:200
anti-SFTPC	WRAB-76694	Seven Hills Bioreagent	1:200
anti-CC10	sc-365992	SantaCruz	1:200
anti-acTUB	T7451	Sigma-Aldrich	1:200

Supplementary Table S2. List of the primers used in this study.

Genes	Primer (Forward)	Primer (Reverse)
Gastric organoid markers		
GAPDH	GAA GGT GAA GGT CGG AGT C	GAA GAT GGT GAT GGG ATT TC
SOX2	GCT TAG CCT CGT CGA TGA AC	AAC CCC AAG ATG CAC AAC TC
PDX1	CGT CCG CTT GTT CTC CTC	CCT TTC CCA TGG ATG AAG TC
HNF6	TGT TGC CTC TAT CCT TCC CA	GGA GGA TGT GGA AGT GGC T
HNF1β	TCA CAG ATA CCA GCA GCA TCA GT	GGG CAT CAC CAG GCT TGT A
MUC6	CAG CAG GAG GAG ATC ACG TTC AAG	GTG GGT GTT TTC CTG TCT GTC ATC
TFF2	TCT GAG ACC TCC ATG ACG C	ATG GAT GCT GTT TCG ACT CC
MUC5AC	CCA AGG AGA ACC TCC CAT AT	CCA AGC GTC ATT CCT GAG
TFF1	AAT TCT GTC TTT CAC GGG GG	GGA GAA CAA GGT GAT CTG CG
GKN1	AGC TAG GGC AGG AGC TAG AAA	GCT TGC CTA CTC CTC TGT CC
GHRL	GCT GGT ACT GAA CCC CTG AC	GAT GGA GGT CAA GCA GAA GG
SST	GCG CTG TCC ATC GTC CTG GCC C	AGC CGG GTT TGA GTT AGC AGA T
GAST	CAG AGC CAG TGC AAA GAT CA	AGA GAC CTG AGA GGC ACC AG
SOX9	GGA GAG CGA GGA GGA CAA GTT C	TTG AAG ATG GCG TTG GGG G
ATP4A	AGT ACG TCA AGT TCG CGA GG	GCA ATG AGA GCG ATT GCC AG
ATP4B	CAG GGG TAA CCT TAA GGC CG	GCA GGA GAA CTT GGT GTG GT
MIST1	CTC ACG CTG GCC AAG AAC TA	GTG GAT CTG CGT GGA GTA CC
PGC	TCT AGG TCC ACT GCT GAC CC	CGG CCC AGC TTG TTA CTT TTC
Cardiomyocyte markers		
HAND2	GAC CCA GGA CTC CGG AAA AG	ACG GGA GTG TCC TCT TCG TA
GATA4	GCC TCT CGG TGT GAC GAG T	AGC ATT GAG CAA AGG GCT CTA
NKX2.5	CAA CTT CGT GAA CTT CGG CG	GGA CTC AGG GTC ATG TTG GG
cTnT	AGG GAG AGC AGA GAC CAT GT	TTT GGA CTC CTC CAT TGG GC
MYL2	AGG CGG AGA GGT TTT CCA AG	GGA CCA CTC TGC AAA GAC GA
MYH7	CAG GAC CTG GTA GAC AAG CTG	ATT CAA GCC CTT CGT GCC AAT
MYL7	GGA GTT CAA AGA AGC CTT CAG C	AAA GAG CGT GAG GAA GAC GG
MYH6	ACC TGT CCA AGT TCC GCA AG	AGG TTG GCA AGA GTG AGG TT
Lung bud organoid markers		
NANOG	CAA AGG CAA ACA ACC CAC TT	ATT GTT CCA GGT CTG GTT GC
GATA4	TCC AAA CCA GAA AAC GGA AG	CTG TGC CCG TAG TGA GAT GA
SOX17	CGC TTT CAT GGT GTG GGC TAA GGA CG	TAG TTG GGG TGG TCC TGC ATG TGC TG
SOX2	AGA ACC CCA AGA TGC ACA AC	ATG TAG GTC TGC GAG CTG GT
NKX2.1	GAC ACC ATG AGG AAC AGC G	CTC ATG TTC ATG CCG CTC
SOX9	GTA CCC GCA CTT GCA CAA C	GTG GTC CTT CTT GTG CTG C
FOXJ1	CAA CTT CTG CTA CTT CCG CC	CGA GGC ACT TTG ATG AAG C
SCGB1A1	ATG AAA CTC GCT GTC ACC CT	GTT TCG ATG ACA CGC TGA AA
SFTA3	TGG TGT TCC AAA TAT TGC AGA	GTT CAT CCG AGG CCA AGA
SFTPA1	CAG ACG GGA CCC CTG TAA AC	CCT GTC ATT CCA CTG CCC A
SFTPB	GAG CCG ATG ACC TAT GCC AAG	AGC AGC TTC AAG GGG AGG A
LYZ	AAA ACC CCA GGA GCA GTT AAT	CAA CCC TCT TTG CAC AAG CT
