# Supplementary material for: Development of a quantitative prediction algorithm for target organ-specific similarity of human pluripotent stem cell-derived organoids and cells
Source: Nat Commun. 2021 Jul 23;12:4492. doi: 10.1038/s41467-021-24746-w (PMC8302568; doi:10.1038/s41467-021-24746-w)
Supplement: Supplementary file 2 — Description of Additional Supplementary Files [file 41467_2021_24746_MOESM2_ESM.docx]

Description of Additional Supplementary Files

Title: Supplementary Dataset 1.

Description: List of organ-GEPs (LuGEP, StGEP, HtGEP)
