# Supplementary material for: Development of a quantitative prediction algorithm for target organ-specific similarity of human pluripotent stem cell-derived organoids and cells
Source: Nat Commun. 2021 Jul 23;12:4492. doi: 10.1038/s41467-021-24746-w (PMC8302568; doi:10.1038/s41467-021-24746-w)
Supplement: Supplementary file 4 — Reporting Summary [file 41467_2021_24746_MOESM4_ESM.pdf]

nature research

Corresponding author(s): Hyun-Soo Cho

Last updated by author(s): Jun 21, 2021

Reporting Summary

Nature Research wishes to improve the reproducibility of the work that we publish. This form provides structure for consistency and transparency in reporting. For further information on Nature Research policies, see our Editorial Policies and the Editorial Policy Checklist.

Statistics

For all statistical analyses, confirm that the following items are present in the figure legend, table legend, main text, or Methods section.

n/a	Confirmed	
☐	☒	The exact sample size (*n*) for each experimental group/condition, given as a discrete number and unit of measurement
☐	☒	A statement on whether measurements were taken from distinct samples or whether the same sample was measured repeatedly
☐	☒	The statistical test(s) used AND whether they are one- or two-sided<br>*Only common tests should be described solely by name; describe more complex techniques in the Methods section.*
☒	☐	A description of all covariates tested
☐	☒	A description of any assumptions or corrections, such as tests of normality and adjustment for multiple comparisons
☐	☒	A full description of the statistical parameters including central tendency (e.g. means) or other basic estimates (e.g. regression coefficient) AND variation (e.g. standard deviation) or associated estimates of uncertainty (e.g. confidence intervals)
☐	☒	For null hypothesis testing, the test statistic (e.g. F, t, r) with confidence intervals, effect sizes, degrees of freedom and P value noted<br>*Give P values as exact values whenever suitable.*
☒	☐	For Bayesian analysis, information on the choice of priors and Markov chain Monte Carlo settings
☒	☐	For hierarchical and complex designs, identification of the appropriate level for tests and full reporting of outcomes
☒	☐	Estimates of effect sizes (e.g. Cohen's *d*, Pearson's *r*), indicating how they were calculated

Our web collection on statistics for biologists contains articles on many of the points above.

Software and code

Policy information about availability of computer code

Data collection	A total of 8,555 RNA-Seq data (transcript RPKM of GTEx analysis version 6) of 53 tissues were obtained through the publicly available GTEx database to select genes that could reflect the characteristics and functions of each tissue.
Data analysis	(1) Organ-algorithm and panel; For characterization tissue specific genes, the data was analyzed through t-test and confidence intervals of R (Version 3.6.0). And we used the Manhattan distance in R to construct the score representing differentiation of organoids.<br>(2) FACS analysis : FlowJo V10 software<br>(3) Single cell analysis : , we performed principal component analysis (PCA) and then applied t-distributed stochastic neighbor embedding (tSNE) with 10 principal components (PCs) for visualization. Violin plots were generated by Loupe Cell Browser v5.0 with typical tissue-specific markers

For manuscripts utilizing custom algorithms or software that are central to the research but not yet described in published literature, software must be made available to editors and reviewers. We strongly encourage code deposition in a community repository (e.g. GitHub). See the Nature Research guidelines for submitting code & software for further information.

Data

Policy information about availability of data

All manuscripts must include a data availability statement. This statement should provide the following information, where applicable:

- Accession codes, unique identifiers, or web links for publicly available datasets
- A list of figures that have associated raw data
- A description of any restrictions on data availability

All sequencing data are deposited in NCBI GEO (GSE178858)

Field-specific reporting

Please select the one below that is the best fit for your research. If you are not sure, read the appropriate sections before making your selection.

[x] Life sciences [ ] Behavioural & social sciences [ ] Ecological, evolutionary & environmental sciences

For a reference copy of the document with all sections, see nature.com/documents/nr-reporting-summary-flat.pdf

Life sciences study design

All studies must disclose on these points even when the disclosure is negative.

Sample size	To construct organ specific gene panel and algorithm, we used 8,555 samples in 53 tissues from GTEx database
Data exclusions	No data were excluded from the analysis
Replication	All attempts at replication were successful. (1) for validation of algorithm, we produced RNA-seq data with total RNA from 20 tissues purchased from Clontech (Human Total RNA Master Panel) and confirmed the similarity using organ panels and algorithms. Also, we used TCGA (normal live lung and stomach) and GEO (normal heart; GSE133054) sample data to calculate organ similarity by the Organ-GEP algorithms. (2) All experiment were performed in three independent experiments for replication.
Randomization	Samples were grouped according to human organs & organoids
Blinding	The investigators were not blinded during data collection as the biological groupswere well defined and handled in parallel. Computational analysis was performed by data scientists different from the researchers

Reporting for specific materials, systems and methods

We require information from authors about some types of materials, experimental systems and methods used in many studies. Here, indicate whether each material, system or method listed is relevant to your study. If you are not sure if a list item applies to your research, read the appropriate section before selecting a response.

Materials & experimental systems

n/a	Involved in the study	
[ ]	[x]	Antibodies
[ ]	[x]	Eukaryotic cell lines
[x]	[ ]	Palaeontology and archaeology
[x]	[ ]	Animals and other organisms
[x]	[ ]	Human research participants
[x]	[ ]	Clinical data
[x]	[ ]	Dual use research of concern

Methods

n/a	Involved in the study	
[x]	[ ]	ChIP-seq
[ ]	[x]	Flow cytometry
[x]	[ ]	MRI-based neuroimaging

Antibodies

Antibodies used	anti-FOXA2 (07-633,Millipore,1:100),anti-SOX17 (MAB1924,R&D system,1:50),anti-HNF1β (sc-7411,SantaCruz,1:100),anti-PDX1 (AF2419,R&D system,1:50),anti-SOX2 (MAB2018,millipore,1:100),anti-KLF5 (ab137676,abcam,1:100),anti-SOX9 (sc-20095,SantaCruz,1:50),anti-SST (A0566,Dako,1:200),anti-MUC5AC (ab78660,Abcam,1:200),anti-MUC6 (sc-33668,SantaCruz,1:50) anti-ECAD (610182,BD biosciences,1:200),anti-ECAD (AF648,R&D system,1:200),anti-cTnT (Ab64623,abcam,1:100),anti-MYL2 (10906-1-AP,Proteintech,1:50),anti-NKX2.5 (AF2444,R&D,1:100),anti-MLC2a (311011,Synoptic system,1:100),anti-cTnT-PE (564767,BD,1:40),anti-Mouse IgG1-PE (130-092-212,Miltenyi Biotec,1:40),anti-NKX2.1 (Ab76013,Abcam,1:200), anti-SOX9 (AB5535,Millipore, 1:200),anti-EPCAM (2626,Cell Signaling,1:200),anti-SFTPC (WRAB-76694,Seven Hills Bioreagent,1:200) anti-CC10 (sc-365992,SantaCruz,1:200),anti-acTUB (T7451,Sigma-Aldrich,1:200)
Validation	Validated by manufacturers<br>(1) anti-FOXA2 (07-633,Millipore,1:100); Species Reactivity: Human, Mouse, Rat; Application: IHC, WB<br>(2) anti-SOX17 (MAB1924,R&D system,1:50); Species Reactivity: Human; Application: WB, ChIP, ICC<br>(3) anti-HNF1β (sc-7411,SantaCruz,1:100); Species Reactivity: Human, Mouse, Rat, Zebrafish; Application: WB, ICC, IHC<br>(4) anti-PDX1 (AF2419,R&D system,1:50); Species Reactivity: Human ; Application: WB, IHC, ICC<br>(5) anti-SOX2 (MAB2018,millipore,1:100) ; Species Reactivity: Human, Mouse, Rat ; Application: WB, CyTOF, ICC, Flow cytometry<br>(6) anti-KLF5 (ab137676,abcam,1:100) ; Species Reactivity: Mouse, Human ; Application: IHC, WB, ICC<br>(7) anti-SOX9 (sc-20095,SantaCruz,1:50) ; Species Reactivity: human, mouse; Application:WB, IHC<br>(8) anti-SST (A0566,Dako,1:200) ; Species Reactivity: human, mouse, rat, zebrafish; Application: WB, IHC, ICC, Flow cytometry<br>(9) anti-MUC5AC (ab78660,Abcam,1:200) ; Species Reactivity: Human ; Application: IHC, ICC<br>(10)anti-MUC6 (sc-33668,SantaCruz,1:50) ; Species Reactivity: Human ; Application:WB, ICC, IHC, IP

(11)anti-ECAD (610182,BD biosciences,1:200) ; Species Reactivity: Human, Mouse, Rat, Dog ; Application: WB, IP, ICC, IHC
(12)anti-ECAD (AF648,R&D system,1:200); Species Reactivity: Human/Mouse ; Application: WB, CyTOF, ICC, Flow cytometry, IHC
(13)anti-cTnT (Ab64623,abcam,1:100) ; Species Reactivity: Human ; Application: WB, ICC, Flow cytometry, IHC, ELISA
(14)anti-MYL2 (10906-1-AP,Proteintech,1:50) ; Species Reactivity: human, mouse, rat, zebrafish ; Application: IF, IHC, IP, WB,ELISA
(15)anti-NKX2.5 (AF2444,R&D,1:100) ; Species Reactivity: Human; Application: WB, IHC
(16)anti-MLC2a (311011,Synoptic system,1:100) ; Species Reactivity: human, rat, mouse ; Application: WB, ICC, Flow cytometry, IHC
(17)anti-cTnT-PE (564767,BD,1:40) ; Species Reactivity: Mouse, Human, Rat, Pig, Dog, Chicken, Rabbit, Guinea Pig ; Application: Flow cytometry
(18) anti-Mouse IgG1-PE (130-092-212,Miltenyi Biotec,1:40) ; Target Mouse Control IgG1; Application: Flow cytometry
(19) anti-NKX2.1 (Ab76013,Abcam,1:200) ; Species Reactivity: human, mouse, rat ; Application: WB, ICC, Flow cytometry, IHC, IP
(20) anti-SOX9 (AB5535,Millipore, 1:200) ; Species Reactivity: human, mouse, rat, Chicken ; Application: IHC, WB, ChIP, ChIP-seq, ICC, IF
(21) anti-EPCAM (2626,Cell Signaling,1:200) ; Species Reactivity: human ; Application: WB, ICC, Flow cytometry, IHC, IP
(22) anti-SFTPC (WRAB-76694,Seven Hills Bioreagent,1:200) ; Species Reactivity:Human, mouse ; Application: WB
(23) anti-CC10 (sc-365992,SantaCruz,1:200) ; Species Reactivity: mouse, human, rat ; Application: WB, IP, IHC, ICC
(24) anti-acTUB (T7451,Sigma-Aldrich,1:200) ; Species Reactivity: plant, hamster, rat, mouse, human, pig, monkey, frog, bovine, chicken ; Application: WB, IHC,

Eukaryotic cell lines

Policy information about cell lines

Cell line source(s)	H9 hESCs were purphased from WiCell (https://www.wicell.org/home/ stem-cells/catalog-of-stem-cell-lines/wa09.cmsx). CRL2097 was purchased from ATCC (https://www.atcc.org/products/all/CRL-2097.aspx)
Authentication	We authenticated hESC and hiPSC line by STR array, and it was also confirmed by karyotype analysis that it was maintained as a normal karyotype
Mycoplasma contamination	Cells were tested negative for mycoplasma.
Commonly misidentified lines (See ICLAC register)	No commonly misidentified cell lines were used.

Flow Cytometry

Plots

Confirm that:

- [x] The axis labels state the marker and fluorochrome used (e.g. CD4-FITC).
- [x] The axis scales are clearly visible. Include numbers along axes only for bottom left plot of group (a 'group' is an analysis of identical markers).
- [x] All plots are contour plots with outliers or pseudocolor plots.
- [x] A numerical value for number of cells or percentage (with statistics) is provided.

Methodology

Sample preparation	The differentiated DE(Definitive Endoderm) was dissociated into single cells and incubated with antibody in DPBS containing 2% FBS and 2 mM EDTA at RT for 30 min. After the cells were washed with dPBS, they were analyzed with Accuri C6 flow cytometry (BD Biosciences). For the differentiated CMs(cardiomyocytes), cells were dissociated as single cells and fixed and permeabilized using the Transcription Factor Buffer Set (BD Biosciences, San Jose, CA) according to the manufacturer's instructions. Antibodies were diluted 1:40 and incubated at 4°C for 40 min. After incubation, the samples were washed twice with Perm/Wash Buffer (BD Biosciences).
Instrument	FACS analysis was performed with an Accuri C6 flow cytometer (BD Biosciences)
Software	Data were analyzed using FlowJo V10 software (TreeStar, USA)
Cell population abundance	10000 cells was used for FACS analysis
Gating strategy	No gating was applied the all flow cytometry data

- [ ] Tick this box to confirm that a figure exemplifying the gating strategy is provided in the Supplementary Information.
